# Non-canonical activation of OmpR drives acid and osmotic stress responses in single bacterial cells

**DOI:** 10.1038/s41467-017-02030-0

**Published:** 2017-11-14

**Authors:** Smarajit Chakraborty, Ricksen S. Winardhi, Leslie K. Morgan, Jie Yan, Linda J. Kenney

**Affiliations:** 1 Mechanobiology Institute, National University of Singapore, T-Lab, 5A Engineering Drive 1, Singapore, 117411 Singapore; 2 Department of Physics, National University of Singapore, Singapore, 117551 Singapore; 3 Jesse Brown Veterans Affairs Medical Center, Chicago, IL 60612 USA; 4 Department of Microbiology & Immunology, University of Illinois-Chicago, Chicago, IL 60612 USA; 5 Department of Biochemistry, National University of Singapore, Singapore, 117596 Singapore

## Abstract

Unlike eukaryotes, bacteria undergo large changes in osmolality and cytoplasmic pH. It has been described that during acid stress, bacteria internal pH promptly acidifies, followed by recovery. Here, using pH imaging in single living cells, we show that following acid stress, bacteria maintain an acidic cytoplasm and the osmotic stress transcription factor OmpR is required for acidification. The activation of this response is non-canonical, involving a regulatory mechanism requiring the OmpR cognate kinase EnvZ, but not OmpR phosphorylation. Single cell analysis further identifies an intracellular pH threshold ~6.5. Acid stress reduces the internal pH below this threshold, increasing OmpR dimerization and DNA binding. During osmotic stress, the internal pH is above the threshold, triggering distinct OmpR-related pathways. Preventing intracellular acidification of *Salmonella* renders it avirulent, suggesting that acid stress pathways represent a potential therapeutic target. These results further emphasize the advantages of single cell analysis over studies of population averages.

## Abstract (web summary)

OmpR is a transcription factor activated in acid and osmotic responses of Gram-negative bacteria, leading to acidification of the bacterial cytoplasm. Here the authors use single cell pH imaging to define the role of OmpR-regulated genes in the acidification response to osmotic and acid stress of *Salmonella* and *E. coli*.

## Introduction

Eukaryotes tightly regulate cytoplasmic pH and osmolality to be isosmotic and pH balanced with blood (290 mOsm Kg^−1^, pH 7.35–7.45). In contrast, Gram-negative bacteria can undergo large changes in osmolality (up to 1800 mOsm Kg^−1^)^1^ and endure a substantial reduction in cytoplasmic pH (to 5.5 or lower)^2^. *E. coli* is known to be capable of colonizing the gastrointestinal tract by its ability to grow over a wide pH range (4.5–9.0)^3–5^, yet *E. coli* and *S.* Typhimurium are considered to be neutralophiles, i.e., they maintain their intracellular pH (pH_i_) between 7.5 and 7.7^6^. Previous studies report that in response to acid stress, *E. coli* rapidly acidifies and then recovers^7^, but the essential components of this recovery process remain poorly understood. Furthermore, many intracellular pathogens survive within a membrane-bound acidic compartment inside macrophages. We recently placed a novel FRET DNA biosensor (the “I-switch”) inside *S.* Typhimurium and measured its intracellular pH in the macrophage vacuole^2^. Surprisingly, the *S.* Typhimurium cytoplasm acidified in a process that was dependent on the EnvZ-OmpR signal transduction system and acidification was essential for *S.* Typhimurium virulence.

Because our observation of prolonged acidification of *S.* Typhimurium^2^ was in conflict with previous studies in *E. coli* ^7^, we set out to re-examine pH regulation in *E. coli* and *S*. Typhimurium. We labeled *S.* Typhimurium in culture with either the I-switch, or with the fluorescent pH indicator BCECF-AM. Single cell analysis using either probe indicated that bacteria fail to recover from acid shock, but maintain their cytoplasmic pH to be slightly less acidic (~0.3–0.6 pH units) than the extracellular pH (pH_e_). Acidification is dependent on the response regulator OmpR, known to be involved in the acid tolerance response in *S.* Typhimurium^2^ and in osmoregulation of outer membrane proteins OmpF and OmpC (see ref. ^8^ for a review). In response to both acid and osmotic stress, the cytoplasm acidifies in an OmpR-dependent manner, although the pathways are distinct.

The pathways are distinct because the intracellular pH is more acidic in response to acid stress compared to osmotic stress. Under acidic conditions, the sensor kinase EnvZ is activated by increased helicity of a disordered region that surrounds the phosphorylated histidine^9^. This activation step promotes interaction with OmpR^10^, driving an active OmpR conformation. This is evident by an EnvZ-dependent, phosphorylation-independent increase in OmpR dimer formation, which facilitates high-affinity DNA binding and alteration of gene expression (usually repression). An EnvZ mutant that is unable to interact with OmpR^10^ fails to acidify in response to both acid and osmotic stress. OmpR-driven repression is independent of OmpR phosphorylation and thus, is non-canonical^11^. An acid-dependent increase in the affinity of OmpR binding to DNA at the *ompC* and *cadB/A* promoters was identified by atomic force microscopy (AFM). In contrast, genes that are activated by OmpR (e.g., *ompF* and *ompC*), require OmpR phosphorylation to create a productive interaction with RpoA, the alpha subunit of RNA polymerase^12^.

Herein, we establish that pHluorin, when expressed as an arabinose-inducible, plasmid-encoded, pH-sensitive GFP, is heterogeneous with respect to its expression in single cells and thus, it is not a good indicator for pH measurements in bacteria. Many previous studies also used sodium benzoate as a clamping agent that was reported to equilibrate the internal pH_i_ with the external pH_e_. Comparison of the standard curve of cells clamped using the ionophore nigericin and generated from the I-switch or BCECF, with cells clamped using the weak acid sodium benzoate dramatically illustrates that sodium benzoate is a poor choice as a clamping agent. Lastly, we provide evidence that the *E. coli* strain MC4100 responds differently to extracellular acid or osmotic stress compared to the probiotic Nissle strain^13,14^ and to MG1655, a sequenced strain^15^. It appears that the large deletions that occurred during the construction of MC4100 for making *lacZ* transcriptional fusions made it a poor strain for studying *E. coli* physiology^16^. Thus, we now have a radically different view of pH regulation in Gram-negative bacteria that centers on an acid-sensitive conformational switch in OmpR that facilitates repression of acid response genes to maintain an acidic cytoplasm. Our results also implicate acidification as essential for virulence and we can now identify potential targets for therapeutic intervention. Lastly, this study identifies a pH threshold in bacteria, below which OmpR phosphorylation is low, but dimerization is high and DNA binding is driven by an acid-dependent conformational change in OmpR. Unique genes are expressed above and below this threshold, allowing integration of osmotic and acid stress signals.

In this study, we establish OmpR as a global regulator of acid and osmotic stress in *S.* Typhimurium and *E. coli*. OmpR represses distinct sets of genes based on the intracellular pH threshold (~6.5) that neutralize the bacterial cytoplasm, thus enabling acidification. The response to acid stress involves repression of lysine decarboxylation, whereas the osmotic stress response in *Salmonella* works through repression of *rpoS*, activating an oxidoreductase that produces protons. In *E. coli*, repression of ornithine decarboxylation acidifies the cytoplasm during osmotic stress. OmpR acts non-canonically, in that it depends on its kinase EnvZ, but phosphorylation is not required. Lastly, we highlight the importance of single cell imaging over population averages and explain why our results differ from previous studies.

## Results

### The EnvZ-OmpR acid stress response acidifies the cytoplasm

**Fig. 1:**
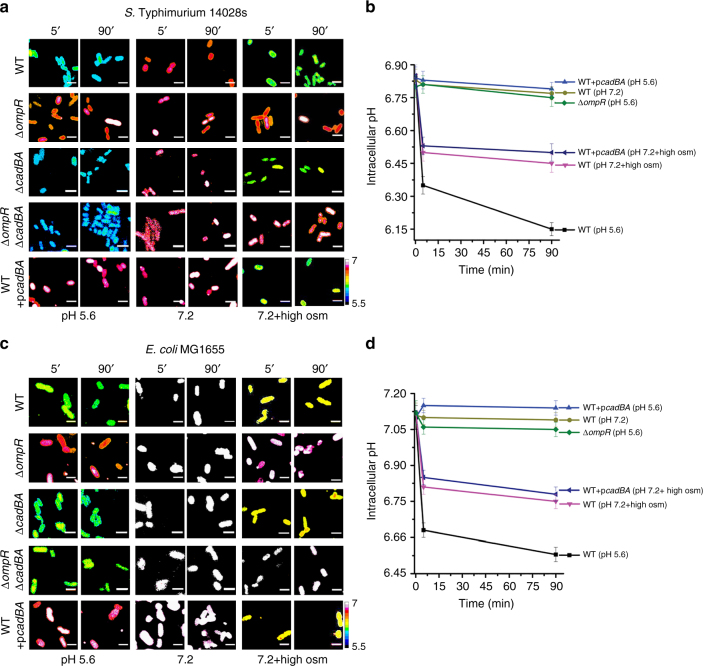
OmpR drives intracellular acidification in response to acid stress. **a**–**d** *S*. Typhimurium and *E. coli* cultures were incubated with 20 µM BCECF for 60 min before imaging. Representative epifluorescence ratio images (R_488/440_) of emission channel 525 nm upon 488 nm excitation and 440 nm excitation were obtained for wild type, an *ompR* null mutant, an *ompR*/cad*BA* null strain, a *cadBA* null strain, and a *cadBA* over-expressed strain of **a** *S*. Typhimurium and **c** *E. coli* incubated at either acidic pH_e_ (5.6) or neutral pH_e_ (7.2) or at pH_e_ (7.2) plus 15% (w/v) sucrose. Using ImageJ software, ratio images were color coded with blue (ratio = 0.1) to white (ratio = 1). Scale bar, 3 µm. A plot of the intracellular pH of 50 cells of wild type and mutants of **b** *S*. Typhimurium and **d** *E. coli* at each indicated time point. Error bars represent the mean ± s.e.m. (*n* = 3). The complete graphs including all strains in **b**, **c** are in Supplementary Fig. 2. Repression of the *cad* operon was not responsible for intracellular acidification during osmotic stress in *S*. Typhimurium and *E. coli*

We previously determined that *S*. Typhimurium reduced its intracellular pH in response to external acid stress in an OmpR-dependent manner^2^, in contrast to previous studies in *E. coli* that reported a rapid recovery within minutes^17,18^. We set out to reconcile these differences by measuring the cytoplasmic pH and recovery of *S.* Typhimurium and *E. coli* immediately following an external acid shift. In *S*. Typhimurium, the cytoplasmic pH decreased from 6.80 to 6.35 within 5 min at pH_e_ 5.6 and reached a plateau value of 6.15 after 90 min (Fig. 1a, b). In contrast, the cytoplasm remained unchanged (pH 6.80) when incubated at pH_e_ 7.2. No decrease in cytoplasmic pH was observed in an *ompR* null strain of *S*. Typhimurium (pH 6.75), confirming the existence of an OmpR-dependent acidification process^2^. In *E. coli*, the initial pH of the cytoplasm was slightly higher (pH 7.13) compared to *S*. Typhimurium (pH 6.80), but a comparable decrease in pH_i_ from 7.13 to 6.55 occurred during 90 min exposure to acid stress (Fig. 1c, d). In an *ompR* null strain of *E. coli*, intracellular pH did not decrease in response to extracellular acid stress, establishing OmpR as a regulator of acid stress in both *S*. Typhimurium and *E. coli* (Fig. 1 and refs. ^2,19^).

### Acidification via OmpR repression of lysine decarboxylation

In *S*. Typhimurium, OmpR prevents recovery from acidification by repressing *cadC/BA*. CadA encodes a lysine decarboxylase, CadB encodes a lysine/cadaverine antiporter, and CadC encodes a transcriptional regulator. We performed quantitative real-time PCR (qRT-PCR) with *E. coli* and observed an increase in transcript levels of *cadB* (4.5-fold) and *cadA* (3-fold) in the *ompR* null mutant, suggesting that *E. coli* OmpR also functions as a repressor at this locus (Supplementary Fig. 1a). OmpR repression resulted from direct interaction at the *cadC/BA* locus, based on electrophoretic mobility shift assays (EMSAs) (Supplementary Fig. 1b). OmpR did not bind at *cadA* (Supplementary Fig. 1b), suggesting that *cadB* and *cadA* are co-transcribed, as we also observed in *S*. Typhimurium^2^.

To determine whether *cadC/BA* repression was sufficient to prevent recovery from acid stress, we measured the response of an *ompR*/*cadBA* null strain, a *cadBA* null strain, and a *cadBA* over-expressed strain of *E. coli* exposed to similar acid stress. As with *S*. Typhimurium (Fig. 1a, b and ref. ^2^), OmpR was no longer required for cytoplasmic acidification when *cadBA* was eliminated, indicating that the CAD system was the main pathway for recovery from acidification between pH 6.1 and 6.5 (Fig. 1c, d). Furthermore, when *cadBA* was over-expressed in *E. coli*, OmpR was unable to repress *cadC/BA*, and the cytoplasm was neutralized.

Overexpression of additional decarboxylases, such as glutamate (*gadBA*), arginine (*speA*), and S-adenosylmethionine decarboxylase (*speD*), during acid stress did not neutralize the pH_i_ (Supplementary Fig. 1c, d), suggesting that *cadBA* was the major system that eliminated protons upon acid stress in *E. coli* over the approximate pH_i_ range of 6.1–6.5. This was not surprising, since the glutamate and arginine decarboxylation systems exhibit much lower pH optima (pH 4 and 5, respectively)^20^, compared to lysine (pH 6.1–6.5)^21^, S-adenosylmethionine decarboxylase (pH 7.4)^22^ and ornithine decarboxylase systems (pH 7.0)^23^. Furthermore, the pH_i_ response of the *cadBA* null strain was similar to the wild type (Fig. 1). Thus, in both *S.* Typhimurium and *E. coli*, OmpR directly repressed *cadC/BA* to enable cytoplasmic acidification under acid stress.

### Osmotic stress acidifies *S.* Typhimurium and *E. coli*

EnvZ has been described as an osmosensor, as high osmolality activates OmpR to differentially upregulate *ompC* and repress *ompF* (reviewed in ref. ^8^). Since EnvZ senses and responds to a concentrated cytoplasm^2,9^, we hypothesized that the cytoplasm might acidify during osmotic stress, indicating that EnvZ is actually functioning as a pH sensor. Using sucrose as the osmolyte, addition of 15% sucrose (w/v; 787 mOsm Kg^−1^) to the medium at pH_e_ 7.2 led to a decrease in the pH_i_ of *S*. Typhimurium from 6.80 to 6.45 (Fig. 1a, b). In contrast, the *ompR* null strain remained near-neutral pH_i_, decreasing by <0.1 pH unit to 6.75 (Fig. 1a, b). A similar decrease was evident in *E. coli*, where osmotic stress reduced the pH_i_ from 7.13 to 6.75. The pH_i_ of the *ompR* null *E. coli* strain did not decrease in response to sucrose-induced osmotic stress and remained at pH_i_ 7.1 (Fig. 1c, d). Thus, in both *S.* Typhimurium and *E. coli*, increasing external osmolality stimulated a prolonged, OmpR-dependent intracellular acidification (Fig. 1).

### Osmolyte-induced acidification is distinct from acid stress

Overexpression of CAD did not protect *S*. Typhimurium or *E. coli* from acidification by osmotic stress (Fig. 1). Furthermore, elimination of *cadB/A* in an *ompR* null strain did not restore intracellular acidification at high osmolality in both *S.* Typhimurium (Fig. 1a, b and Supplementary Fig. 2a) and *E. coli* (Fig. 1c, d and Supplementary Fig. 2b), suggesting osmotic stress and acid stress pathways were distinct. However, osmolyte-driven intracellular acidification was sufficient to trigger virulence gene expression of *Salmonella* pathogenicity island 2 (SPI-2), as evidenced by *ssrB* transcription and effector SseJ secretion (Supplementary Fig. 3a, b). To identify OmpR targets in response to acid stress, we performed a microarray-based expression analysis and compared the transcriptome of *ompR* null strains with wild-type strains of *S.* Typhimurium and *E. coli* during acid or osmotic stress (see data availability for full results through the GEO).

**Fig. 2:**
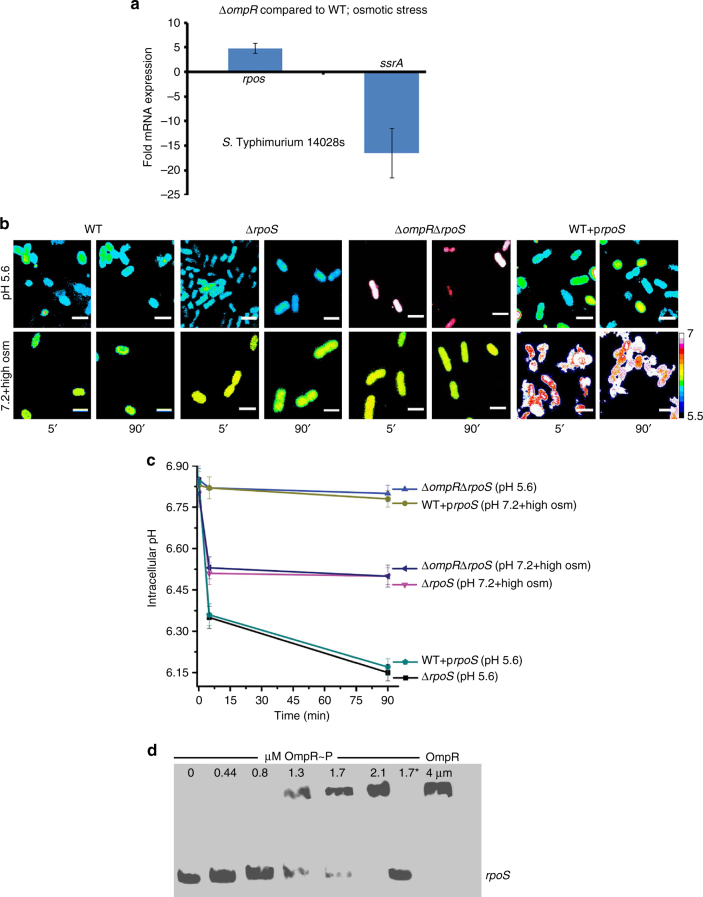
Osmotic stress acidifies *S.* Typhimurium via OmpR repression of *rpoS*. **a** mRNA levels of *rpos* and *ssrA* (a positive control) genes were determined by qRT-PCR from wild-type and *ompR* null strains of *S*. Typhimurium grown at high osmolality. Fold expression changes in the *ompR* null strain was compared to the wild-type level and plotted. The error bars represent the mean ± s.d. (*n* = 6). In the *ompR* null strain, *rpoS* transcription was increased, suggesting OmpR repression. **b** Representative images of R_488/440_ are shown after 5 and 90 min incubation at pH_e_ 7.2 or pH_e_ 7.2 in the presence of 15% (w/v) sucrose (high osm) of wild type, an *ompR*/*rpoS* null strain, an *rpoS* null strain, and an *rpoS* over-expressed strain of *S*. Typhimurium. Scale bar, 3 μm. **c** A plot from 50 cells of the R_488/440_ ratios of various mutants of *S*. Typhimurium at indicated times. Symbols represent the mean ± s.e.m. (*n* = 3). **d** An EMSA examined the interaction between OmpR and *rpoS*. OmpR was incubated with 10 fmol of biotin end-labeled *rpoS*. Lane 1.7* contained 100-fold excess unlabeled DNA

In our *S*. Typhimurium microarray, the alternate sigma factor *rpoS* was upregulated 4.4-fold in the absence of *ompR*. RpoS was implicated in the acid-inducible exponential-phase acid tolerance response (ATR) of *S*. Typhimurium^24–26^ and was essential for survival under acid stress^27,28^. In agreement with the upregulation observed by microarray, the *rpoS* transcript was up 4.8-fold in the *ompR* mutant (Fig. 2a), suggesting that OmpR functions as a repressor at *rpoS*. Furthermore, the *rpoS* over-expressed strain maintained near-neutral pH_i_ (6.8) in response to osmolytes, but was fully capable of cytoplasmic acidification by acid stress (Fig. 2b, c and Supplementary Fig. 4). Thus, the responses to acid stress and osmotic stress were distinct. Similarly, in the absence of *rpoS*, OmpR was not required for intracellular acidification at high osmolality (Fig. 2b, c and Supplementary Fig. 4). In response to acid stress, OmpR repressed the *cadC/BA* operon to maintain an acidic cytoplasm, whereas at high osmolality, an *rpoS*-dependent pathway was involved in *S*. Typhimurium acidification (Figs. 1 and 2). OmpR acted directly at *rpoS*, as determined by EMSAs with purified OmpR protein (Fig. 2d), i.e., OmpR directly repressed *rpoS* transcription at high osmolality.

**Fig. 3:**
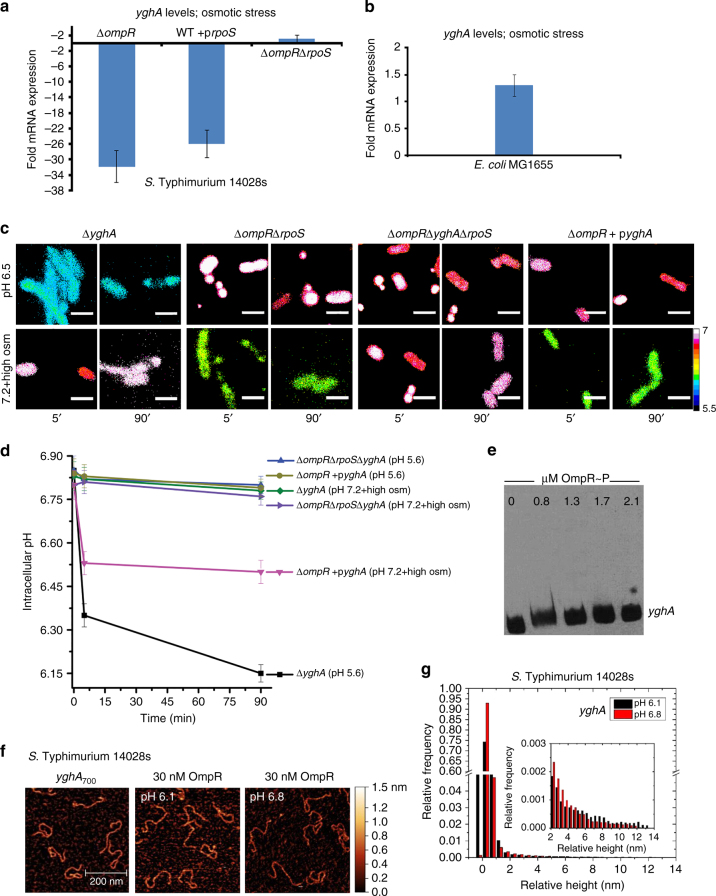
OmpR relieves RpoS repression of *yghA* during osmotic stress. **a** mRNA levels of *yghA* at pH_e_ 7.2 in the presence of 15% sucrose (w/v) were determined by qRT-PCR from wild type, an *ompR* null strain, an *rpoS* over-expressed strain, and an *ompR/rpoS* null strain of *S*. Typhimurium. Fold expression changes were compared to the wild-type level. Error bars represent the mean ± s.d. (*n* = 6). **b** mRNA of *yghA* at pH_e_ 7.2 in the presence of 15% sucrose (w/v) of an *E. coli ompR* null strain was compared to the wild-type level. **c** Representative images of R_488/440_ are shown after 5 and 90 min incubation at pH_e_ 5.6, or pH_e_ 7.2 + 15% (w/v) sucrose of a *yghA* null strain, an *ompR*/*rpoS* null strain, an *ompR/rpoS/yghA* null strain, and an *ompR* null strain of *S.* Typhimurium with *yghA* over-expressed. Scale bar, 3 μm. **d** Plot of the R_488/440_ ratios of various mutants of *S.* Typhimurium at each indicated time. Data set is represented as the mean ± s.e.m. (*n* = 3). The complete graph, including all strains is in Supplementary Fig. 4. **e** EMSAs were performed with biotin end-labeled *yghA* and OmpR. **f** AFM images of *yghA*_700_ from *S*. Typhimurium with 30 nM OmpR at either acid pH (6.1) or neutral pH (6.8). **g** Relative height distribution histogram of the *yghA* promoter complexed with 30 nM OmpR at either acidic or neutral pH

To identify *rpoS* targets involved in intracellular acidification, we examined the microarray results for candidate targets that were downregulated in the *ompR* null strain and were known to be *rpoS*-dependent. We identified *yghA*, encoding a putative oxidoreductase, which was down 39-fold in the *ompR* null strain at high osmolality compared to the wild-type *S.* Typhimurium. This was confirmed by qRT-PCR, which indicated comparable downregulation of a *yghA* transcript (32-fold) in the *ompR* null strain, as well as in the *rpoS* over-expressed strain (26-fold), implicating RpoS as a repressor of *yghA* (Fig. 3a). In contrast, in *E. coli*, YghA was not involved in osmotic stress-driven acidification, as *yghA* levels of the wild type and an *ompR* null strain were comparable (Fig. 3b). As an oxidoreductase, YghA is predicted to oxidize NADH to liberate H^+^, leading to cytoplasmic acidification. In the absence of YghA, the *S.* Typhimurium cytoplasm would fail to acidify, due to a reduction in acid production. The pH_i_ of a *yghA* null strain was 6.8 (i.e., not acidified) and thus, OmpR was no longer required to repress *rpoS* (Fig. 3c, d). This result suggests a mechanism whereby OmpR relieves *rpoS-*dependent repression of *yghA* (which would be activating), to produce and maintain an acidified cytoplasm in response to osmotic stress. Furthermore, when *yghA* was over-expressed, the pH of the cytoplasm decreased to 6.45. In contrast, the *ompR/rpoS/yghA* null strain maintained near-neutral pH_i_ (Fig. 3c, d). OmpR did not bind directly to the *yghA* promoter, as evident from the EMSA (Fig. 3e). This result was further supported by AFM imaging, and the relative height distribution histogram (Fig. 3f). Instead, OmpR directly repressed *rpoS* transcription (Fig. 2), which increased *yghA* transcription (Fig. 3a, c, d).

Measurements of NAD^+^/NADH were consistent with these results (Supplementary Fig. 5a, b). In the wild-type strain, *yghA* transcription increased 32-fold compared to an *ompR* null strain. Intracellular levels of NAD^+^ in the wild type (52.3 nM) or an *ompR/rpoS* (51.3 nM) null strain were higher than an *ompR* null strain (29.6 nM), a *yghA* null strain (37.3 nM) or an *ompR*/*yghA*/*rpoS* (35.6 nM) null strain grown at high osmolality (Supplementary Fig. 5a, b). Overexpression of *yghA* in an *ompR* null background completely restored intracellular NAD^+^ to wild-type levels (49.6 nM). Increased NAD^+^ levels were only observed at high osmolality and not during acid stress, further emphasizing the distinct responses to acid and osmotic stress.

### OmpR-dependent acidification occurs at high osmolality

**Fig. 4:**
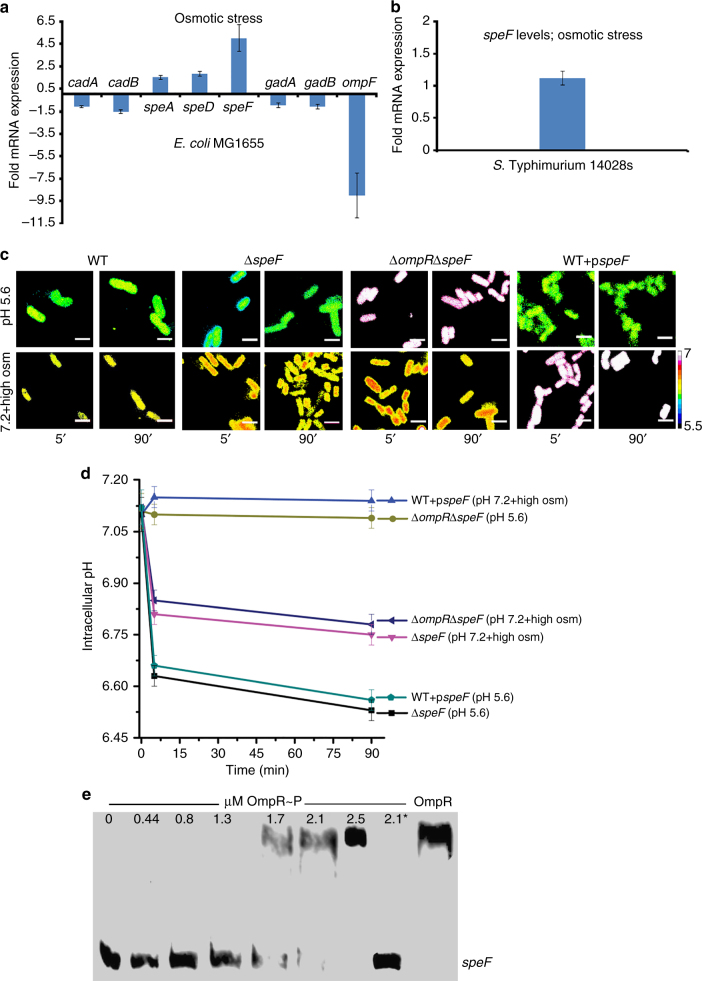
OmpR represses *speF* to acidify *E. coli* during osmotic stress. **a** mRNA levels at pH_e_ 7.2 containing 15% (w/v) sucrose of *cadA, cadB, speA, speD, speF, gadA, gadB*, and *ompF* genes were determined by qRT-PCR from wild-type and *ompR* null strains of *E. coli* MG1655. Fold expression changes were compared to the wild-type level. The error bars represent the mean ± s.d. (*n* = 6). The *ompR* null strain showed increased transcription of *speF* at high osmolality. **b** *speF* mRNA level of an *ompR* null strain of *S*. Typhimurium was compared to the wild-type level at pH_e_ 7.2 in the presence of 15% sucrose (w/v). **c** Representative R_488/440_ images of wild type, an *ompR* null strain, an *ompR*/*speF* null strain, a *speF* null strain, and the *speF* over-expressed strain of *E. coli* grown at pH_e_ 5.6 or pH_e_ 7.2 in the presence of 15% (w/v) sucrose at the indicated time points. Scale bar, 3 μm. **d** Plot of the R_488/440_ ratios of various mutants of *E. coli*. The complete graph including all strains is in Supplementary Fig. 6. Data set is represented as the mean ± s.e.m. (*n* = 3). **e** EMSAs were conducted with biotin end-labeled *speF* to detect OmpR–*speF* interaction. Lane 2.1* contained 100-fold excess unlabeled DNA

Microarray analysis comparing the wild-type and *ompR* null *E. coli* strains indicated that decarboxylase genes were differentially expressed under acid and osmotic stress conditions (Fig. 4a and Supplementary Fig. 1d). To identify the precise pathways, we compared mRNA levels of genes encoding glutamate decarboxylase (*gadB* and *gadA*), arginine decarboxylase (*speA*), S-adenosylmethionine decarboxylase (*speD*), and ornithine decarboxylase (*speF*) by qRT-PCR in the wild-type and *ompR* null mutant (Fig. 4a and Supplementary Fig. 1a). The transcript for *speF* (fivefold) was the most upregulated, compared to *speA* (1.5-fold) and *speD* (1.8-fold) in the *ompR* null strain at high osmolality, suggesting a repressive role of OmpR (Fig. 4a). In *S*. Typhimurium, *speF* levels were identical between the wild-type and *ompR* null strain, indicating the differing response by OmpR in *S*. Typhimurium (RpoS repression of *yghA*) and *E. coli* (*speF*) in response to osmotic stress (Figs. 3 and 4b).

Following osmotic stress, the *cadBA* (Fig. 1c, d), *gadBA*, *speA*, and *speD* overexpression strains all acidified to an extent indistinguishable from wild type, whereas the strain over-expressing *speF* did not acidify (Fig. 4c, d and Supplementary Figs. 1c, d and 6). This result identified *speF* as the predominant decarboxylase that functioned to eliminate protons during osmotic stress to maintain pH homeostasis in *E. coli*. Overexpression of *speF* in response to acid stress did not affect the cellular response to acid stress (Fig. 4c, d and Supplementary Fig. 6), emphasizing again that the acid and osmotic pathways were distinct. As expected, OmpR was not required for acidification in the absence of *speF*, and the *speF* null strain remained similarly acidified compared to the wild type. OmpR bound to the *speF* regulatory region, as evident from an EMSA (Fig. 4e). Thus, in *E. coli*, OmpR represses the *speF* system to prevent recovery from acidification during osmotic stress.

### Phosphorylation of EnvZ-OmpR is pH sensitive

**Fig. 5:**
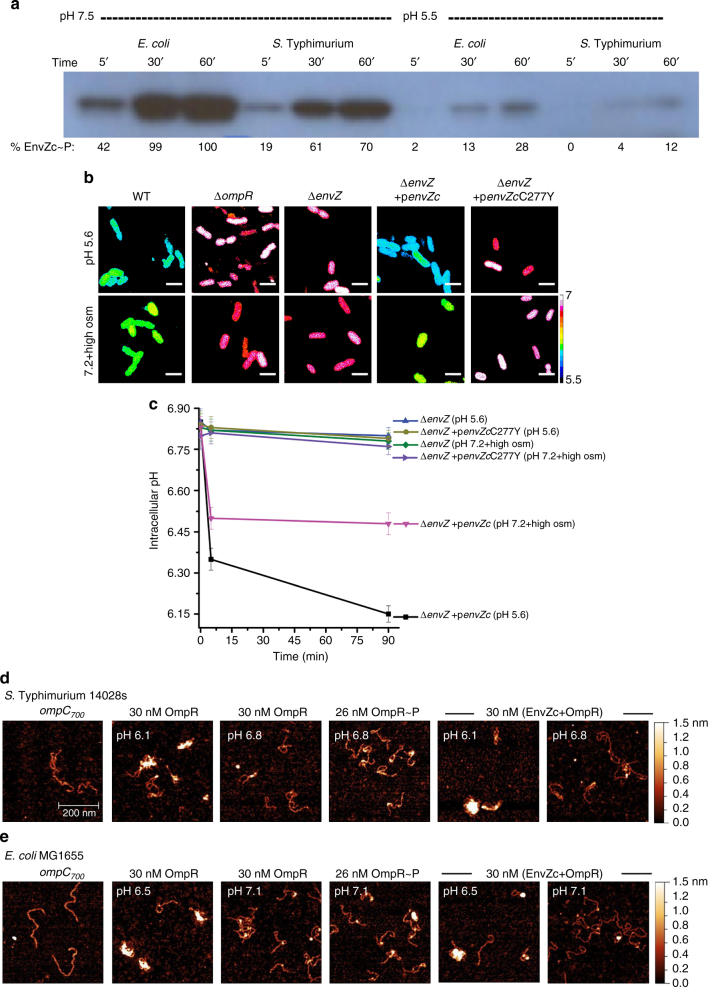
OmpR regulation of intracellular pH is non-canonical. **a** Phosphorylation of EnvZc is acid sensitive. Phosphorylation of *E. coli* EnvZc compared with *S.* Typhimurium EnvZc at pH 7.5 and 5.5. The indicated reaction times are above the panel and the percent phosphorylation (determined by densitometry) is listed below. **b** Representative epifluorescence ratio images (R_488/440_) were obtained for wild type, an *ompR* null, an *envZ* null strain, and the *envZ* null complemented with *envZc* (encoding the cytoplasmic domain of EnvZ), and an *envZ* null complemented with *envZcC277Y* in *S*. Typhimurium. Cells were incubated at either acidic pH_e_ 5.6 or pH_e_ 7.2 in the presence of 15% (w/v) sucrose. Scale bar, 3 µm. **c** A plot of the intracellular pH of 50 cells of wild-type and *S*. Typhimurium mutants at each indicated time point. Error bars represent the mean ± s.e.m. (*n* = 3). **d**, **e** OmpR binding to DNA is pH sensitive. AFM images of the 700 bp *ompC* promoter (*ompC*_*700*_) from **d** *S*. Typhimurium and **e** *E. coli* with 30 nM OmpR at acid pH and at neutral pH (the pH used was from the measured values in Fig. 1), 30 nM OmpR~P (phosphorylated by acetyl phosphate), and 30 nM (EnvZc + OmpR) at different pH_e_ values. The lengths of the *ompC* promoter in *S*. Typhimurium and *E. coli* were similar (Supplementary Fig. 9h, i)

When the cytoplasm was acidified from either acid or osmotic stress, OmpR was “activated”, leading to repressive effects on transcription at *cadC/BA*, *speF*, or *rpoS* (Figs. 1–4). We suspected that EnvZ-OmpR phosphorylation might be pH sensitive, as the histidine phosphorylation site must be unprotonated to act as a nucleophile toward ATP^29^. *S*. Typhimurium or *E. coli* EnvZ was phosphorylated with [γ-^32^P]-ATP and the percentage of EnvZ~P was determined by densitometry (Fig. 5a). EnvZ phosphorylation in *S*. Typhimurium was less robust (70%) than *E. coli* EnvZ (100%). At pH 5.5, EnvZ phosphorylation was substantially lower when compared to phosphorylation at pH 7.5 for *E. coli* (28%) and *S*. Typhimurium (12%), raising the question as to how does activation of OmpR occur during acid and osmotic stress?

### Acidification does not require OmpR phosphorylation

Canonically, response regulators are phosphorylated by their cognate kinases and phosphorylation drives dimerization. The phosphorylated dimer is an active complex that binds DNA with high affinity and interacts with RNA polymerase to activate transcription. However, some orphan response regulators that lack cognate kinases activate transcription without phosphorylation by mimicking an active interface via dimerization^30^; (see ref. ^11^ for a recent review). We reasoned that there might be an acid-dependent conformational change in OmpR that was independent of phosphorylation. We also knew from our previous measurements that EnvZc had higher affinity for OmpR compared to OmpR~P^31,32^, which would promote an interaction. We measured the cytoplasmic pH of *S*. Typhimurium in response to acid and osmotic stress in a D55A OmpR mutant that was incapable of phosphorylation^33^ in the presence of the EnvZ kinase. Cytoplasmic acidification occurred in the D55A mutant background in response to either acid or osmotic stress, indicating that OmpR phosphorylation was not required for acidification (Supplementary Fig. 7a, b). Intriguingly, this phosphorylation-independent activation was completely dependent on the presence of the EnvZ kinase^2^. We therefore examined whether an EnvZ mutant that was incapable of interacting with OmpR (EnvZc C277Y)^10^ was still capable of acid-dependent OmpR repression (Fig. 5b, c). In the presence of the EnvZc C277Y mutant, the *S.* Typhimurium cytoplasm was no longer acidified in response to acid stress (Fig. 5b, c). Thus, OmpR must interact with EnvZc to repress CAD, even though phosphoryl transfer was not involved.

Gel filtration profiles of EnvZc, EnvZc C277Y, OmpR, (OmpR + EnvZc), and (OmpR + EnvZc C277Y) established that in isolation, both wild-type EnvZc and the EnvZc C277Y mutant existed as dimers, whereas OmpR was monomeric (Supplementary Fig. 8a, b). In the presence of wild-type EnvZc, OmpR addition generated a higher molecular weight complex, representing two OmpR molecules bound to an EnvZc dimer (Supplementary Fig. 8a). OmpR dimerization upon interaction with an EnvZc dimer is in agreement with our previous findings using fluorescence cross-correlation spectroscopy^10^. Addition of OmpR to the EnvZc C277Y mutant generated profiles that were identical to the individual proteins in isolation, i.e., no interacting complex was evident (Supplementary Fig. 8b) and ref. ^10^. OmpR is unusual among response regulators, because it can bind DNA in the absence of phosphorylation^34^, presumably because some percentage of the OmpR population exists in an activated, dimeric state. We thus examined whether the oligomeric state of OmpR was affected by acidic conditions (Supplementary Fig. 8c). The percentage of OmpR dimers increased substantially (to 63–73%), compared to neutral pH, where dimers were undetectable (Supplementary Fig. 8c). Thus, acid pH can also drive an OmpR conformational change that results in unphosphorylated OmpR dimers. In vivo, this process is driven by interaction with its kinase EnvZ.

### Acid pH stimulates OmpR binding to DNA

How does acidification affect OmpR/DNA interactions? Most DNA-binding assays are sensitive to acidic pH^34,35^. For this reason, we turned to AFM to visualize OmpR interactions with the *ompC* promoter of *S.* Typhimurium (Fig. 5d) and *E. coli* (Fig. 5e) or the *cadBA* promoter (Supplementary Fig. 9). OmpR was added to a solution buffered to an identical pH_i_ that we measured during acid stress (Fig. 1), indicated in the panels. Acid pH alone did not stimulate OmpR aggregation, as evident in the panels that contain OmpR protein in the absence of DNA (Supplementary Fig. 9a). Addition of OmpR at pH 6.1 (*S*. Typhimurium) led to an increase in binding to DNA, compared to addition of OmpR at pH 6.8 (Fig. 5d and Supplementary Fig. 9b). At pH 6.8, a localized binding of OmpR to *ompC* DNA was visible as specific, discrete foci (see Supplementary Fig. 9d for quantitation of binding and other controls). Addition of OmpR~P prepared from acetyl phosphate phosphorylation was in-between the level of binding observed at pH 6.8 and 6.1 (Fig. 5d). A similar stimulation of OmpR binding to DNA in acidic conditions was also evident in *E. coli* (Fig. 5e and Supplementary Fig. 9c, f), although the level of acidification was less (6.5 vs. 6.1). In the presence of EnvZ, acidic conditions further increased OmpR binding to the *ompC* promoter compared to OmpR alone (Fig. 5d, e; right panels, and Supplementary Fig. 9e, g). The simplest interpretation of this result is that in vivo, acid pH promotes an activating conformation of EnvZ^9,36^ that promotes interaction with OmpR, inducing an OmpR conformational change that stimulates dimer formation and favors OmpR binding to DNA. This process is stimulated by OmpR contact with EnvZ, in an interaction that does not involve phosphorylation. Thus, an EnvZ- and acid-dependent conformational change is sufficient to stimulate OmpR binding to DNA (see “Discussion” section).

### How to reconcile our results with previous studies

Our results with the I-switch and BCECF-AM in *E. coli* and *S.* Typhimurium (this work and ref. ^2^) differ from previously published studies on the response of *E. coli* ^18,37^ and *S.* Typhimurium^38,39^ to acid stress using pHluorin, a pH-sensitive fluorophore. In addition to using a different fluorophore, some measurements were performed in media with poor buffering capacity using HCl to initiate acid stress. We lowered the pH_e_ of M63 media supplemented with casein hydrolysate from 7.5 to 5.5 by adding 8.5 mM HCl and measured the pH_i_ of *E. coli* using a similar acid-induction strategy, but measured the response with BCECF-AM and fluorescence microscopy as before. In both *S*. Typhimurium and *E. coli*, the cytoplasm was acidified, indicating that the differing results were not a result of different stress conditions (Supplementary Fig. 10a, b).

### Plasmid expression of pHluorin is heterogeneous

**Fig. 6:**
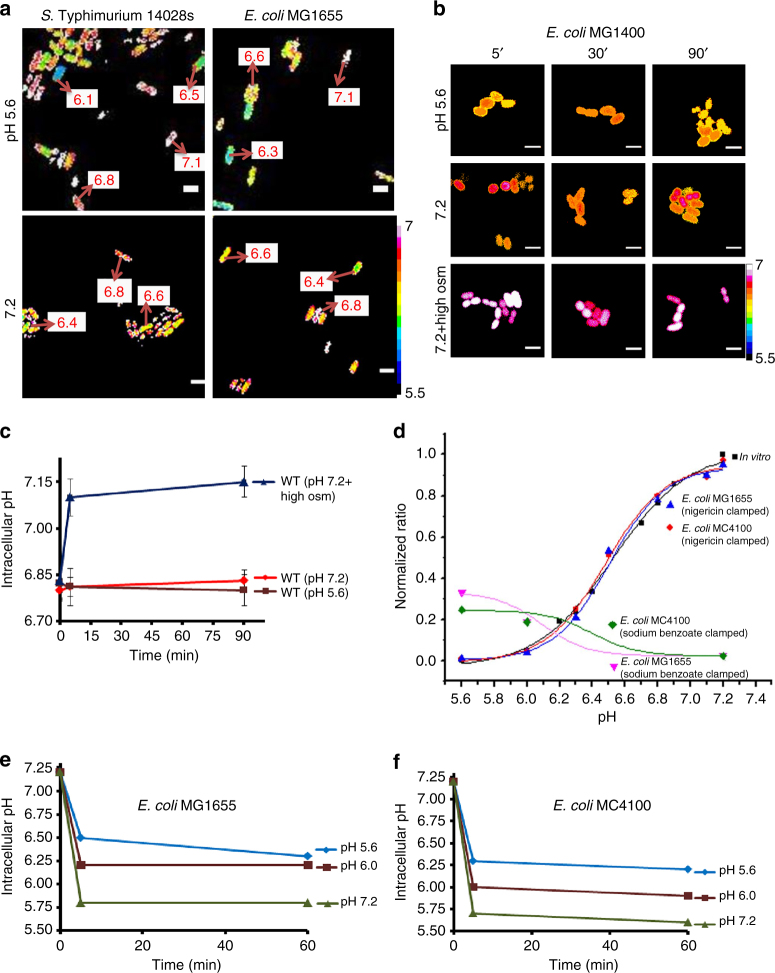
Spurious results from using pHluorin, MC4100, and clamping agent sodium benzoate. **a** *S.* Typhimurium and *E. coli* strains harboring a plasmid containing pH-sensitive *gfp* (pHluorin) exhibit heterogeneity in pH_i_. Representative epifluorescence (R_405/488_) ratio images of emission channel 525 nm upon 405 nm excitation and 488 nm excitation were obtained for wild-type cultures at either acidic pH_e_ 5.6 or neutral pH_e_ 7.2. Using ImageJ software, the ratio images were color coded with blue (ratio = 0.1) to white (ratio = 1). Scale bar, 3 µm. The pH of individual cells after 30 min is indicated in the panel. **b** Representative epifluorescence ratio images (R_488/440_) were obtained for wild-type cultures of *E. coli* MC4100 incubated at pH_e_ 5.6, pH_e_ 7.2, and pH_e_ 7.2 with 15% (w/v) sucrose at the indicated times. Scale bar, 3 µm. **c** A plot of the intracellular pH of 50 cells incubated under acid and osmotic stress conditions at each indicated time. Error bars represent mean ± s.e.m. (*n* = 3). **d** Cells were clamped by either 40 μM nigericin or 30 mM sodium benzoate and the R_488/440_ ratio intensities were plotted as a function of pH and overlaid on their in vitro calibration curve. Intracellular pH values determined from BCECF flluorescence of **e** *E. coli* MG1655 and **f** MC4100 clamped with sodium benzoate at pH_e_ 5.6, pH_e_ 6.1, and pH_e_ 7.2 were plotted

We next examined whether differences were due to the use of different fluorophores. Most previous studies measured the average fluorescence of cultures in solution, whereas our measurements were performed in single cells. We transformed *S.* Typhimurium and *E. coli* with ratiometric pHluorin (pH-sensitive GFP) and examined single cells by confocal microscopy after acid or osmotic shock (Fig. 6a). It was immediately apparent that the cells were extremely heterogeneous with respect to pH values in both *S.* Typhimurium and *E. coli*. In fact, in many of the cells, there was no apparent response to acid stress, while in others, there was acidification, but to varying levels. If one determined the average pH of this population in solution, it might very well appear to have “recovered” from acid stress. This level of heterogeneity was not observed in single cells using BCECF-AM (this work) or using the I-switch^2^. Thus, expressing pHluorin using the arabinose-inducible pBAD promoter is not a good indicator for measuring intracellular pH, because it is not uniformly distributed^40^. Constitutive expression of pHluorin was homogeneously distributed, but the fluorescence signal was weak, as previously reported^41^.

### MC4100 does not acidify during acid or osmotic stress

Another explanation for the differences might be that different strains were employed. We used the sequenced *E. coli* strain MG1655^15^, whereas many previous studies used MC4100^17^. We measured the response of MC4100 to acid stress, using our approach (Fig. 6b, c). MC4100 was slightly acidified initially compared to MG1655 (pH 6.80 vs. 7.13, respectively), which was comparable to the pH_i_ of *S*. Typhimurium (Figs. 1 and 6). In response to pH_e_ 5.6, *E. coli* MC4100 maintained its cytoplasmic pH (pH_i_ = 6.83) throughout the experiment (Fig. 6b, c). The pH_i_ remained essentially unchanged whether the pH_e_ was 7.2, 5.6, or induced by 15% (w/v) sucrose (Fig. 6b, c and Supplementary Fig. 10a, b). In contrast, addition of osmolytes actually increased the pH_i_ (from 6.8 to 7.15), as reported^18^. This result was opposite to what we observed with *E. coli* MG1655. Cytoplasmic acidification in the non-pathogenic *E. coli* Nissle 1917, a widely used probiotic strain^14^, was comparable to *E. coli* MG1655 and *S*. Typhimurium (Supplementary Fig. 10d).

### Sodium benzoate is not a good clamping agent

Most significantly, previous studies generated a standard curve using uncouplers that were reported to collapse ΔpH^6,17,18,38,39^. Sodium benzoate was commonly employed for this purpose. Unfortunately, sodium benzoate was not effective at setting pH_e_ = pH_i_ (Fig. 6d); the standard curve differed dramatically from curves generated from nigericin clamping. To examine this issue further, we used BCECF-AM to measure the pH_i_ of *E. coli* MG1655 in response to various external pH conditions in the presence of 30 mM sodium benzoate. It was evident that at pH_e_ = 5.6, the pH_i_ was 6.3; at pH_e_ = 6.0, the pH_i_ was 6.2 and at pH_e_ = 7.2, the pH_i_ was 5.8 (Fig. 6e). Thus, using sodium benzoate at neutral pH_e_, the intracellular pH would be presumed to have been neutralized, when it was actually quite acidic. These values were not strain dependent, i.e., they were similar for MC4100 and MG1655 (Fig. 6e, f). Thus, it was evident that sodium benzoate was not an effective clamping agent. This finding also explains why previous measurements of *S.* Typhimurium pH were substantially different from ours^38,39^ and why *E. coli* was reported to return to a neutral pH_i_^6,17,18^ after an acid stress. More recent measurements in *S.* Typhimurium using our methods (although not performed in single cells)^42^ now are in keeping with our previous findings^2^.

## Discussion

**Fig. 7:**
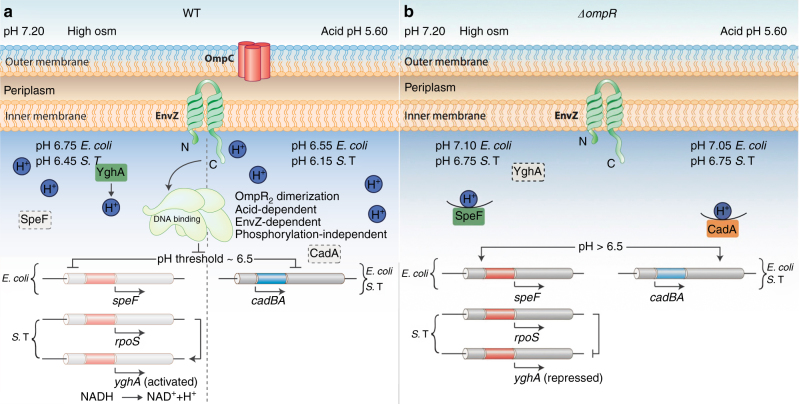
Model for OmpR-driven acidification in response to acid and osmotic stress. Under acid and osmotic stress conditions, both *S*. Typhimurium and *E. coli* are acidified in an OmpR-dependent manner. OmpR requires interaction with EnvZ, but not phosphorylation to bring about cytoplasmic acidification. **a** In wild-type bacteria, under acid stress, OmpR represses the *cad* operon to eliminate proton consumption, resulting in acidification. At high osmolality, in *S*. Typhimurium, OmpR represses *rpoS* to relieve *rpoS* repression of *yghA*, producing protons. In *E. coli*, OmpR represses ornithine decarboxylase (*speF)*, enabling cytoplasmic acidification. The pH optima of CadA is 6.1–6.5^21^, this optima contributes to a threshold of response. At pH 6.5 and below (achieved during acid stress), OmpR represses the *cad* operon, resulting in acidification. At high osmolality (pH 6.75 *E. coli*, 6.45, *S.* Typhimurium), acidification is less, because the CAD system is working to restore neutrality. Acidification occurs through proton production (*S.* Typhimurium) or repression of a different amino acid decarboxylation system (*E. coli*). Intracellular acidification is required for activating SPI-2-dependent effector secretion. EnvZ senses and responds to cytoplasmic acidification via helix-coil transitions. A physical interaction of OmpR with EnvZ drives a conformational change, resulting in unphosphorylated OmpR dimer formation. EnvZ-dependent OmpR dimerization creates an active OmpR_2_ interface, favoring DNA binding and subsequent repression. **b** In the *ompR* null strain, during acid stress, CadC/BA is expressed, and drives amino acid decarboxylation. This process consumes intracellular protons, restoring cytoplasmic pH and maintaining intracellular pH homeostasis. At high osmolality, in an *ompR* null *S*. Typhimurium strain, RpoS represses *yghA*, preventing proton release, resulting in a neutralized cytoplasm. In an *ompR* null *E. coli* strain, activated SpeF eliminates cytoplasmic protons during ornithine decarboxylation, maintaining pH homeostasis

The results presented herein establish a new paradigm for pH regulation in Gram-negative bacteria such as *E. coli* and *S*. Typhimurium (Fig. 7). In contrast to the prevailing view, we determined that *E. coli* and *S*. Typhimurium do not recover (within 90 min) due to the repressive actions of OmpR. In both *S*. Typhimurium and *E. coli*, OmpR represses the CAD system to acidify the cytoplasm during acid stress (Fig. 1). In *S*. Typhimurium, the pathway for acidification during osmotic stress involves *rpoS* and the putative oxidoreductase YghA. Increasing YghA expression (via OmpR repression of *rpoS*), increased proton production, which acidified the cytoplasm. In *E. coli*, OmpR repressed *speF* (which consumes protons) during osmotic stress (Fig. 5 and model, Fig. 7).

Our results indicate that EnvZ functions to sense acidic pH in the cytoplasm, resulting from extracellular acid or osmolytes (Figs. 1 and 2). Although OmpR was capable of repressing genes in the absence of phosphorylation, EnvZ was required. We interpret this result in the following way: acidification promotes helicity^36^; EnvZ responds to cytoplasmic signals by increased helicity of the 17 amino acid region surrounding the phosphorylated histidine^9^. Phosphorylation was slow at acid pH (Fig. 5a), but a physical interaction of OmpR with EnvZ promoted dimerization (Fig. 5b, c and Supplementary Fig. 8a, b), creating an active OmpR_2_ interface that bound DNA with higher affinity (Fig. 5d, e). With an isoelectric point of OmpR at 6.04, its conformation is exquisitely sensitive to changes in cytoplasmic acidity.

The present work identifies a critical pH threshold in bacteria that establishes a signaling node. In *Salmonella*, acidification to pH 6.1 leads to OmpR repression of CAD (Fig. 1) and activation of SPI-2 gene expression, as evident by increasing *ssrB* transcription and SseJ effector secretion (Supplementary Fig. 3). *E. coli* is acidified to pH 6.55, again via OmpR repression of CAD. Osmolytes also lead to acidification, but only to ~pH 6.45 (*Salmonella*) or 6.75 (*E. coli*) (Fig. 1). The cytoplasm is less acidic during osmotic stress due to the competing effects of CAD activation (Fig. 4) and relief of *rpoS* repression leading to *yghA* activation in *Salmonella*, or *speF* repression in *E. coli*. This threshold likely is set by the level of OmpR “activation” and perhaps the effects of acid pH on its conformation. The activated state of OmpR is influenced by numerous factors, including a physical interaction with EnvZ (Fig. 5b, c and Supplementary Fig. 8a, b), phosphorylation^35,43^, dimerization (Supplementary Fig. 8a, b, c), and an acid-dependent effect on OmpR conformation (Fig. 5d, e). We are presently exploring the effect of acid pH on OmpR conformation by NMR to determine whether effects of acid pH differ from or are similar to the effect of EnvZ on OmpR conformation.

Most significantly, the present work highlights that the method for setting pH_e_ = pH_i_ using sodium benzoate actually produces the opposite effect. At a neutral external pH, addition of sodium benzoate substantially acidified both *S.* Typhimurium and *E. coli* (Fig. 6). This finding, we believe, accounts for the substantial differences between our studies and the published literature. This study provides evidence for a new view of the response to acid/osmotic stress in bacteria and emphasizes the intriguing differences in prokaryotes compared to eukaryotes, where intracellular pH and osmolality are tightly regulated. A deeper understanding of these differences will provide new approaches for combating the virulence strategies employed by pathogens to survive in acid environments and secrete virulence factors.

## Methods

### Bacterial strains, antibodies, and growth conditions

*S enterica*. serovar Typhimurium 14028s and *E. coli* MG1655 were used in this study unless otherwise indicated. To determine the acid and osmotic stress response, bacterial strains were grown in a modified N-minimal medium (MgM) buffered with 100 mM Tris (pH 7.2 ± 15% (w/v) sucrose) or 100 mM MES (pH 5.6) as described^2^. The casamino acids included in the media provided sufficient lysine for activation of the CAD system. The following antibodies were used for immuno-labeling experiments: rabbit polyclonal anti-HA (catalog no. S190–108, Axil Scientific) 1:5000, anti-rabbit secondary antibody (catalog no. SC-2357, Santa Cruz) 1:5000. Ampicillin was used at 100 µg ml^−1^, chloramphenicol at 30 µg ml^−1^, kanamycin 30 µg ml^−1^, and tetracycline at 12.5 µg ml^−1^. For clamping experiments, bacteria were incubated at various external pH values with either nigericin (40 µM) or sodium benzoate (30 mM) at RT for 1 h. Overnight wild-type cultures of *S.* Typhimurium and *E. coli* in LB-expressing pHluorins were subcultured into fresh LB in the presence of 0.2% L-arabinose for 2 h (OD ~0.25). The cultures were then centrifuged and incubated in MgM media at either acidic pH_e_ 5.6 or neutral pH_e_ 7.2 containing 0.2% L-arabinose. The intracellular pH was measured every 30 min for 3 h (OD ~0.8).

### Wide-field fluorescence and confocal microscopy

For pH measurements using BCECF-AM, wide-field images were collected using an Olympus IX71 Inverted Microscope (Applied Precision DeltaVision Deconvolution microscope system) equipped with ×100, 1.4 numerical aperture objective lens. Image capture was performed with the CoolSnap HQ, a fast, high-resolution, high-quantum efficiency, cooled CCD camera (Photometrics CoolSNAP HQ2 (CCD); 1392 × 1040 pixels; 11 fps). Two channels were selected for acquiring paired sets of images corresponding to emission wavelength (525/36 nm) upon donor excitation of either 490 ± 20 nm (pH-sensitive wavelength), or 436 ± 10 nm (pH-insensitive wavelength). Both channels had the same exposure time of 10 s. The emission ratios upon excitation (490/436) were obtained by using the ratio plus plug-in of ImageJ software version 1.42. The ratios were plotted as a function of pH, and were comparable to the in vitro calibration curve. The high and low values were color coded and calibrated to their respective pH values as described in ref. ^2^.

For pH measurements using pHluorin, confocal imaging was conducted on an inverted Nikon A1Rsi-Polarizing Module equipped with CFI Plan ApochromatVC ×100, 1.4 numerical aperture objective lens using Coherent CUBE lasers at 405 and 488 nm excitation with set appropriate dichroics. Sequential scans were performed to obtain paired set of images by using excitation wavelengths of either (405 ± 30 nm) or (470 ± 40 nm) and the emission was recorded through a 525/50 nm band pass filter on a Andor DU897 EMCCD camera. The ratiometric images were obtained by dividing the emission at excitation 405 by 470 nm and analyzed using the ratio plus plug-in of ImageJ version 1.42. High and low values were color coded and calibrated to their respective pH values.

### Measurement of BCECF in *S*. Typhimurium and *E coli*

Cultures of *S*. Typhimurium and *E. coli* were pre-incubated with 20 μM BCECF-AM for 60 min before the shift to acidic pH_e_ (5.6), neutral pH_e_ (7.2), or high osmolality pH_e_ 7.2 plus 15% (w/v) sucrose. Cell-permeant BCECF incorporation into cells is evident from the fluorescent images. Cells were then washed with the incubation media to eliminate extracellular BCECF prior to imaging. Ratios of fluorescence intensities of the emission channel (525 nm) upon 488 nm excitation and 440 nm excitation were obtained and compared to the in vitro standard curve as described above and in ref. ^2^. Cells were placed on microscope slides (Marienfeld) coated with 1% (w/v) agarose and images were analyzed by ImageJ version 1.42.

### Construction of mutants and overexpression strains

Chromosomal copies of *yghA* and *rpoS* from wild-type or an *ompR* null strain of *S*. Typhimurium and *cadBA*, *speF* from wild-type or an *ompR* null strain of *E. coli* were replaced by *tetRA* using λ-Red recombination techniques^44^. To generate an *ompR/yghA/rpoS* null strain of *S.* Typhimurium, a Cm^R^ cassette amplified from plasmid pKD3 using primers *yhgA:*:*Cm* #1F and *yhgA:*:*Cm* #1R was integrated into an *ompR/rpoS* double null strain. The *rpoS*, *speA*, *speD, speF*, *gadBA*, *yghA*, and *cadBA* over-expressed strains were generated by cloning the genes into pBR322 by replacement of *bla* ^2^. The pH-sensitive *gfp* (“pHluorin”) gene was cloned into plasmid pMPM*-*A5omega and placed under control of the *arabinose*-inducible pBAD promoter. OmpR site-specific mutant D55 (primers D55AF/D55AR) and EnvZ site-specific mutant C277Y (primers C277YF/C277YR) were introduced following the QuikChange® Site-Directed Mutagenesis Kit (Stratagene) and substitutions were verified by DNA sequencing. The strains are listed in Table 1 and the primers used are in Supplementary Table 1.

**Table 1:** Strains and plasmid used in this study

**Strain**	Reference	Description
*Salmonella enterica* serovar Typhimurium		
∆*ompR*	^47^	NA
*cadBA*::Km	^2^	∆*cadBA*
∆*ompR*; *cadBA*::Km	^2^	∆*ompR*∆*cadBA*
*envZ::*Cm	^2^	∆*envZ*
*ssaJ::tetRA*	^2^	∆*ssaJ*
*rpoS*::*tetRA*	This paper	∆*rpoS*
∆*ompR*; *rpoS*::*tetRA*	This paper	∆*ompR*∆rpoS
*rpoS*::*tetRA*; *yghA::*Km	This paper	∆*rpoS∆yghA*
∆*ompR*; *rpoS*::*tetRA*; *yghA::*Km	This paper	∆*ompR*∆*rpoS∆yghA*
*yghA*::*tetRA*	This paper	∆*yghA*
*E. coli* MG1655		
∆*ompR*	^45^	NA
*cadBA*::*tetRA*	This paper	∆*cadBA*
∆*ompR; cadBA*::*tetRA*	This paper	∆*ompR*∆*cadBA*
*speF*::*tetRA*	This paper	∆*speF*
∆*ompR; speF*::*tetRA*	This paper	∆*ompR*∆*speF*
*E. coli* MC4100	ATCC	ATCC
*E. coli* Nissle 1917	ATCC	ATCC
Plasmid		
pKD46	^48^	NA
pWSK29	^49^	NA
pMPM-A5Ω	^50^	NA
pBR322	^51^	NA
pKD3	^48^	NA
pH-sensitive GFP cloned in pMPM-A5Ω	This paper	pHluorin
p*sseJ*-HA	^2^	NA
pBR322-*cadBA* (*S.* Typhimurium 14,028s)	^2^	p*cadBA*
pBR322-*cadBA* (*E*. coli MG1655)	This paper	p*cadBA*
pBR322-*gadBA* (*E*. coli MG1655)	This paper	p*gadBA*
pBR322-*speA* (*E*. coli MG1655)	This paper	p*speA*
pBR322-*speF* (*E*. coli MG1655)	This paper	p*speF*
pBR322-*speD* (*E*. coli MG1655)	This paper	p*speD*
pBR322-*yghA* (*E*. coli MG1655)	This paper	p*yghA*
pBR322-*rpoS* (*E*. coli MG1655)	This paper	p*rpoS*
pWSK29-*ompR*	^2^	p*ompR*
pWSK29-*ompR* D55A	This paper	P*ompR*D55A
pET28b-EnvZc (*E*. coli MG1655)	^45^	NA
pET28b-EnvZc (C277Y) (*S.* Typhimurium 14,028s)	This paper	NA
pET28b-EnvZc (*S.* Typhimurium 14,028s)	This paper	NA
pET15b-OmpR	^45^	NA
pMPM-A5Ω-*envZc*	^2^	p*envZc*
pMPM-A5Ω-*envZc* (C277Y)	This paper	P*envZc*(C277Y)

### Electrophoretic mobility shift assay

EMSAs were performed using the Lightshift chemi-luminescence EMSA kit (Research instruments) according to the manufacturer’s instructions as described^2^. Upstream regions of *E. coli cadB* (347 bp), *cadA* (350 bp), *speF* (384 bp), and *S*. Typhimurium rpoS (420 bp) and *yhgA* (370 bp) were amplified using biotinylated oligonucleotides. Ten fmol of biotinylated DNA was used in a 15 μl reaction containing binding buffer (10 mM Tris, pH 7.5, 50 mM KCl) along with 2.5% (w/v) glycerol, 1 mM MgCl_2_, 0.05% (w/v) Nonidet P-40, and 1 μg poly(dI-dC). This interaction was specific, as addition of a 100-fold excess of unlabeled DNA was added to examine specific interaction (labeled *). OmpR or OmpR~P protein was added at the concentrations indicated, and samples were separated by electrophoresis on 5% non-denaturing acrylamide gels run in 0.5× Tris-acetate buffer with EDTA.

### RNA isolation and qRT-PCR

*E. coli* and *S*. Typhimurium strains were grown in MgM media at pH 5.6, 7.2, and 7.2 with 15% (w/v) sucrose to OD ~0.6 as described^2^. Total RNA was isolated, followed by cDNA synthesis and quantification. The mRNA expression level of the target gene was normalized relative to 16S rRNA.

### Western blot with anti-SseJ antibody

Total and secreted protein fractions were isolated from wild type and an *ssaJ* null strain of *S*. Typhimurium harboring p*sseJ*-HA. Protein samples were separated by 10% SDS-PAGE followed by transfer to a PVDF membrane. The membrane was incubated with anti-HA (1:5000) or anti-GroEL (1:5000) and a secondary anti-HA HRP antibody (1:5000) was used for detection.

### Overexpression and purification: OmpR, EnvZc, and EnvZc C277Y

*E. coli* BL21 (DE3) was used for overproduction of full-length His-OmpR cloned into pET15b as described^34,45^. Purified OmpR was buffer exchanged (i.e., 20 mM Tris with 200 mM NaCl) buffered at pH 6.1, 6.5, 6.8, or 7.2, and the purity was examined by SDS-PAGE. *S*. Typhimurium and *E. coli* EnvZc were subcloned into pET28b, over-expressed and purified as in ref. ^45^. Overexpression and purification of *S.* Typhimurium EnvZc C277Y protein was as described for wild-type EnvZc.

### Atomic force microscopy

Seven hundred base pair regions from *ompC* (−607 to +93 bp), *cadB* (−673 to +27 bp), and *yghA* (−673 to +27 bp) were gel-purified using the QIAquick Gel Extraction Kit (Qiagen). A glutaraldehyde-modified mica surface was prepared as described^46^. Ten nanogram of the regulatory region was incubated with 30 nM OmpR or OmpR~P (prepared by phosphorylation from acetyl phosphate^35^ at pH 5.6 or 7.2 ± 15% (w/v) sucrose for 15 min at RT. This mixture was then deposited on the mica for 15 min. Images were acquired on a Bruker Dimension FastScan AFM system using the tapping mode with a silicon nitride cantilever (FastScan C, Bruker). Raw AFM images were processed using Gwyddion software (http://gwyddion.net/).

### Gel filtration chromatography

Gel filtration chromatography was carried out on a Hiload 16/60 Superdex 75 pg (Amersham Biosciences) and Superose^®^ 12 10/300 GL (Sigma-Aldrich) size exclusion chromatography columns for OmpR and EnvZc, respectively, on an AKTA FPLC system (Amersham Biosciences) using suitable buffers. Two column volumes of running buffer was used to pre-equilibrate, the flow rate was 0.5 ml min^−1^. Eluted peaks were quantified by absorbance at 280 nm and fractions were analyzed by SDS-PAGE.

### Measurement of intracellular NAD^+^/NADH

Intracellular NAD^+^/NADH levels were determined using an NAD^+^/NADH assay kit (ProBioscience Technologies) from *S*. Typhimurium cultures grown for 90 min at acidic pH_e_ (5.6), neutral pH_e_ (7.2), or high osmolality pH_e_ (7.2) plus 15% (w/v) sucrose. Cells were normalized to OD_600_ = 0.8 and total NAD^+^ and NADH levels were determined per ml of bacteria. Intracellular NAD^+^/NADH levels (nM) were converted using NAD^+^ standards of known concentration.

### Statistical analysis

For intracellular ATP, NAD^+^/NADH, and pH measurements, the results are presented as mean ± s.e.m. For quantification of mRNA levels, it is mean ± s.d.

### Data availability

The microarray data that are available have been deposited with the GEO (accession code: GSE106630). All other relevant data are available from the corresponding author on request.

## Electronic supplementary material

Supplementary Information
