# Supplementary material for: Non-canonical activation of OmpR drives acid and osmotic stress responses in single bacterial cells
Source: Nat Commun. 2017 Nov 14;8:1587. doi: 10.1038/s41467-017-02030-0 (PMC5686162; doi:10.1038/s41467-017-02030-0)
Supplement: Supplementary file 1 — Supplementary Information [file 41467_2017_2030_MOESM1_ESM.pdf]

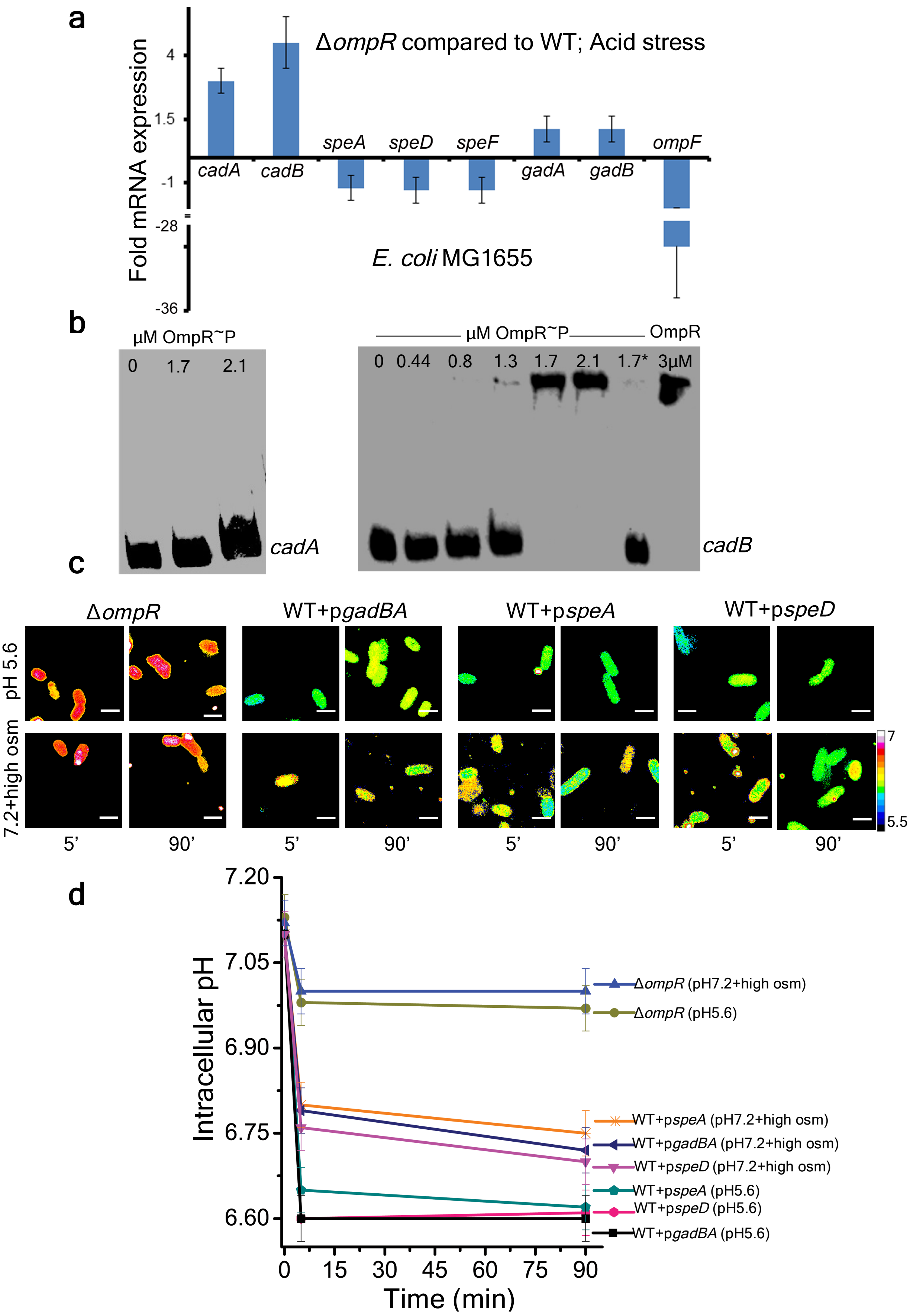
a
Fold mRNA expression
ΔompR compared to WT; Acid stress
cadA
cadB
speA
speD
speF
gadA
gadB
ompF
E. coli MG1655
b
µM OmpR~P
0 1.7 2.1
cadA
µM OmpR~P
OmpR
0 0.44 0.8 1.3 1.7 2.1 1.7* 3µM
cadB
c
ΔompR
WT+pgadBA
WT+pspeA
WT+pspeD
pH 5.6
7.2+high osm
5'
90'
7
5.5
d
Intracellular pH
Time (min)
ΔompR (pH7.2+high osm)
ΔompR (pH5.6)
WT+pspeA (pH7.2+high osm)
WT+pgadBA (pH7.2+high osm)
WT+pspeD (pH7.2+high osm)
WT+pspeA (pH5.6)
WT+pspeD (pH5.6)
WT+pgadBA (pH5.6)

Supplementary Figure 1. Additional OmpR regulated decarboxylase genes in *E. coli* were not responsible for intracellular acidification at high osmolality. **a,** mRNA levels at pH_e 5.6 of *cadA, cadB, speA, speD, speF, gadA, gadB* and *ompF* genes were determined by qRT-PCR from wild-type and *ompR* null strains of *E. coli* MG1655. Fold expression changes in the *ompR* null strain compared to the wild-type were plotted. The error bars represent the mean ± s.d. (n = 6). **b,** EMSA experiments were conducted to examine the interaction of OmpR binding to the *cadA* or *cadB* promoters. OmpR was incubated with 10 fmol of biotin end-labeled *cadA* (left panel) or *cadB* (right panel)**.** The results indicate that OmpR directly binds *cadB* in response to acid stress. Lane 1.7* contained 100-fold excess unlabeled DNA. **c,** Representative images of $R_{480/440}$ were obtained for an *ompR* null mutant and the *gadBA*, *speA*, and the *speD* over-expressed strains of *E. coli* incubated at either acid (pH_e 5.6) or pH_e (7.2) plus 15% sucrose at the indicated time points. The *speA*, *speD* and *gadBA* over-expressed strains were capable of intracellular acidification under both acid and osmotic stress, suggesting other OmpR targets. Scale bar, 3 μm. **d,** A plot of the intracellular pH of fifty cells of the indicated *E. coli* strains. Error bars represent the mean ± s.e.m (n = 3).

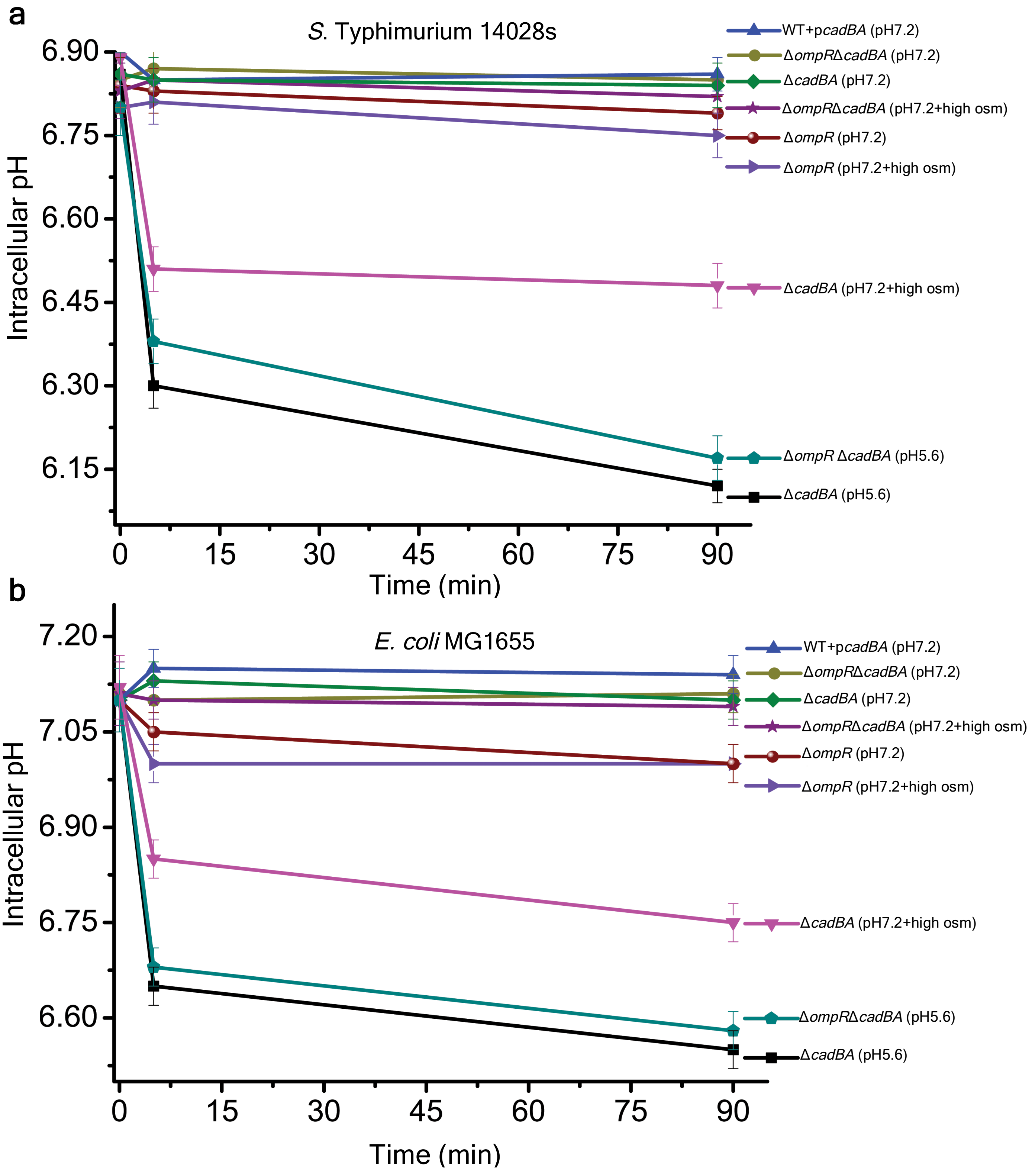

a
S. Typhimurium 14028s
Intracellular pH
6.90
6.75
6.60
6.45
6.30
6.15
0
15
30
45
60
75
90
Time (min)
WT+pcadBA (pH7.2)
ΔompRΔcadBA (pH7.2)
ΔcadBA (pH7.2)
ΔompRΔcadBA (pH7.2+high osm)
ΔompR (pH7.2)
ΔompR (pH7.2+high osm)
ΔcadBA (pH7.2+high osm)
ΔompR ΔcadBA (pH5.6)
ΔcadBA (pH5.6)
b
E. coli MG1655
Intracellular pH
7.20
7.05
6.90
6.75
6.60
0
15
30
45
60
75
90
Time (min)
WT+pcadBA (pH7.2)
ΔompRΔcadBA (pH7.2)
ΔcadBA (pH7.2)
ΔompRΔcadBA (pH7.2+high osm)
ΔompR (pH7.2)
ΔompR (pH7.2+high osm)
ΔcadBA (pH7.2+high osm)
ΔompRΔcadBA (pH5.6)
ΔcadBA (pH5.6)

Supplementary Figure 2. OmpR repression of the *cad* operon is responsible for intracellular acidification in *S.* Typhimurium and *E. coli* during acid stress. The intracellular pH measurements of fifty cells of an *ompR* null mutant, a *cadBA* null strain, an *ompR*/*cadBA* null strain, and a *cadBA* over-expressed strain of **a,** *S*. Typhimurium and **b,** *E. coli* grown at pH_e 5.6, pH_e 7.2 and pH_e 7.2 in the presence of 15% (w/v) sucrose were plotted at each indicated time point. Error bars represent the mean ± s.e.m (n = 3). Intracellular acidification mediated by OmpR at high osmolality was not via repression of the *cad* operon.

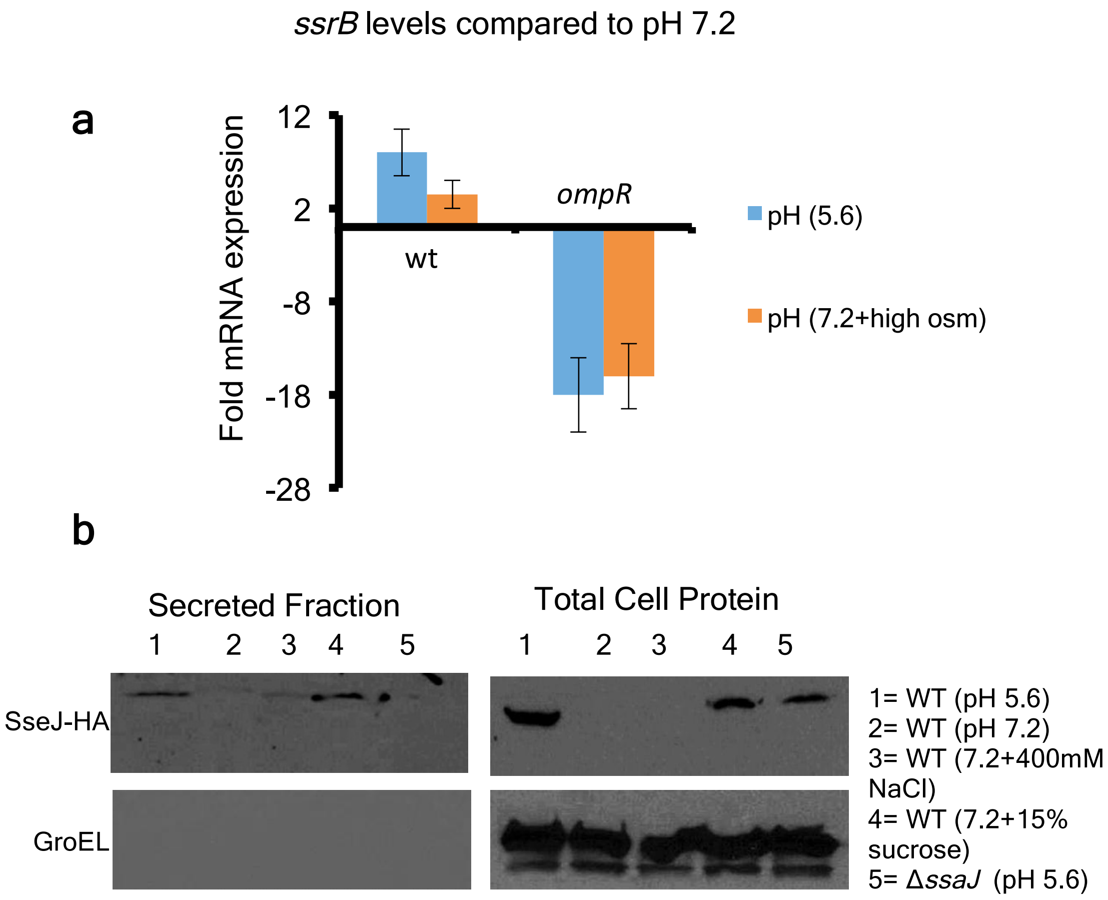

Supplementary Figure 3. Osmotic stress driven intracellular acidification activates SPI-2. a, mRNA levels of *ssrB* at pH$_e$ 5.6 and pH$_e$ 7.2 plus 15% (w/v) sucrose were compared to pH$_e$ 7.2 by qRT-PCR from wild-type and *ompR* null strains of *S*. Typhimurium. Fold expression changes were compared to the wild-type level at pH$_e$ 7.2. The error bars represent the mean ± s.d. (n = 6) **b,** Immunoblot analysis of secreted effector SseJ-HA of whole cell and secreted protein fractions prepared from wild-type and a type III secretion apparatus mutant (*ssaJ* null strain) of *S*. Typhimurium grown at the indicated conditions is shown. Anti-GroEL antibody was used as a loading control.

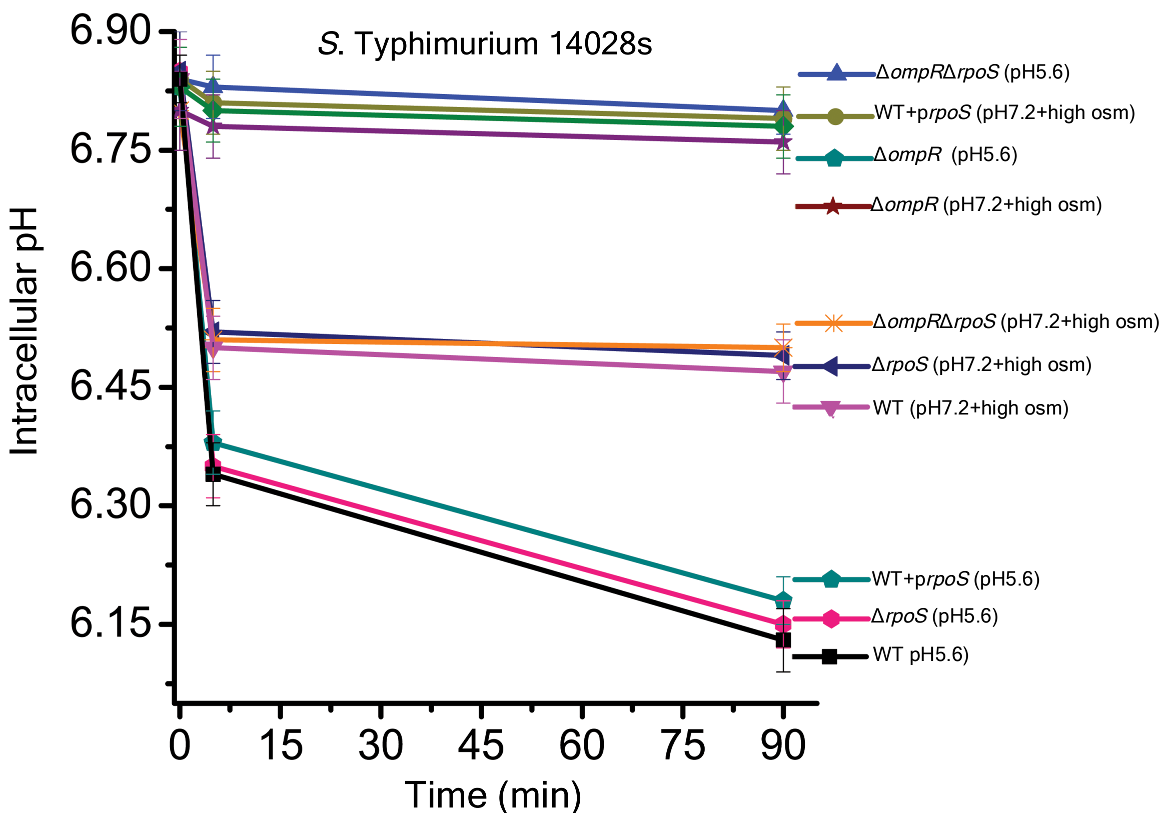

Supplementary Figure 4. Intracellular acidification at high osmolality in *S.* Typhimurium is via OmpR repression of *rpoS*. Plot of intracellular pH measurements of fifty cells of wild-type, an *ompR* null mutant, an *rpoS* null strain, an *ompR*/*rpoS* null strain, and an *rpoS* over-expressed strain of *S.* Typhimurium grown at pH_e 5.6 or pH_e 7.2 plus 15% sucrose were plotted at each indicated time point. Error bars represent the mean ± s.e.m (n = 3).

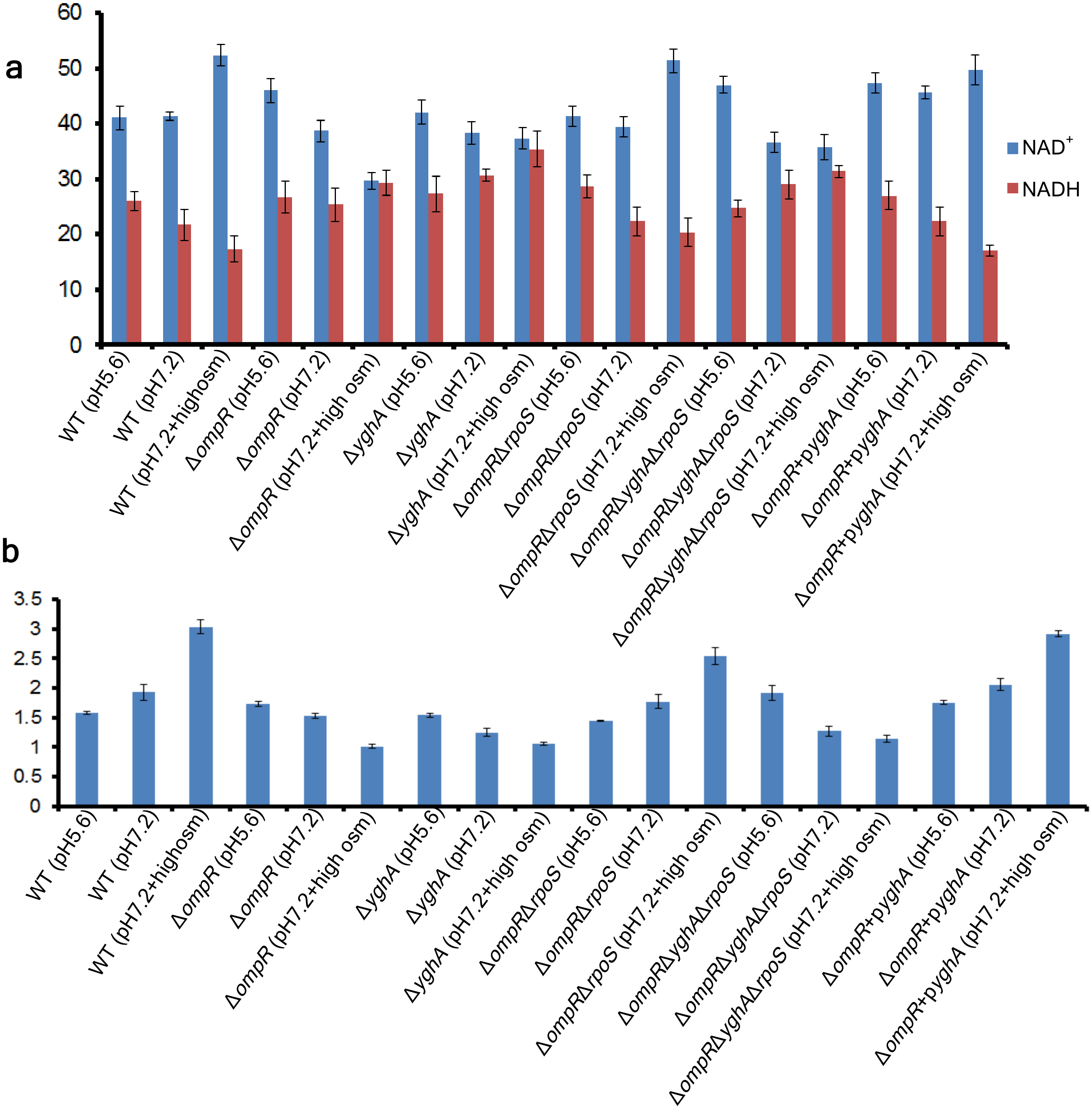
a
60
50
40
30
20
10
0
NAD+
NADH
WT (pH5.6)
WT (pH7.2)
WT (pH7.2+highosm)
ΔompR (pH5.6)
ΔompR (pH7.2)
ΔompR (pH7.2+high osm)
ΔyghA (pH5.6)
ΔyghA (pH7.2)
ΔyghA (pH7.2+high osm)
ΔompRΔrpoS (pH5.6)
ΔompRΔrpoS (pH7.2)
ΔompRΔrpoS (pH7.2+high osm)
ΔompRΔyghAΔrpoS (pH5.6)
ΔompRΔyghAΔrpoS (pH7.2)
ΔompRΔyghAΔrpoS (pH7.2+high osm)
ΔompR+pyghA (pH5.6)
ΔompR+pyghA (pH7.2)
ΔompR+pyghA (pH7.2+high osm)
b
3.5
3
2.5
2
1.5
1
0.5
0
WT (pH5.6)
WT (pH7.2)
WT (pH7.2+highosm)
ΔompR (pH5.6)
ΔompR (pH7.2)
ΔompR (pH7.2+high osm)
ΔyghA (pH5.6)
ΔyghA (pH7.2)
ΔyghA (pH7.2+high osm)
ΔompRΔrpoS (pH5.6)
ΔompRΔrpoS (pH7.2)
ΔompRΔrpoS (pH7.2+high osm)
ΔompRΔyghAΔrpoS (pH5.6)
ΔompRΔyghAΔrpoS (pH7.2)
ΔompRΔyghAΔrpoS (pH7.2+high osm)
ΔompR+pyghA (pH5.6)
ΔompR+pyghA (pH7.2)
ΔompR+pyghA (pH7.2+high osm)

Supplementary Figure 5. OmpR activates *yghA* by eliminating *rpoS*-dependent repression, resulting in increased intracellular NAD$^+$ levels. a, Intracellular NAD$^+$/NADH levels and **b,** NAD$^+$/NADH ratio were determined for the wild-type, an *ompR* null mutant, a *yghA* null strain, an *ompR*/*rpoS* null strain, an *ompR*/*yghA*/*rpoS* and a *yghA* over-expressed strain in an *ompR* null background of *S*. Typhimurium grown for 90 min at the indicated conditions. Error bars represent the mean ± s.e.m (n = 3).

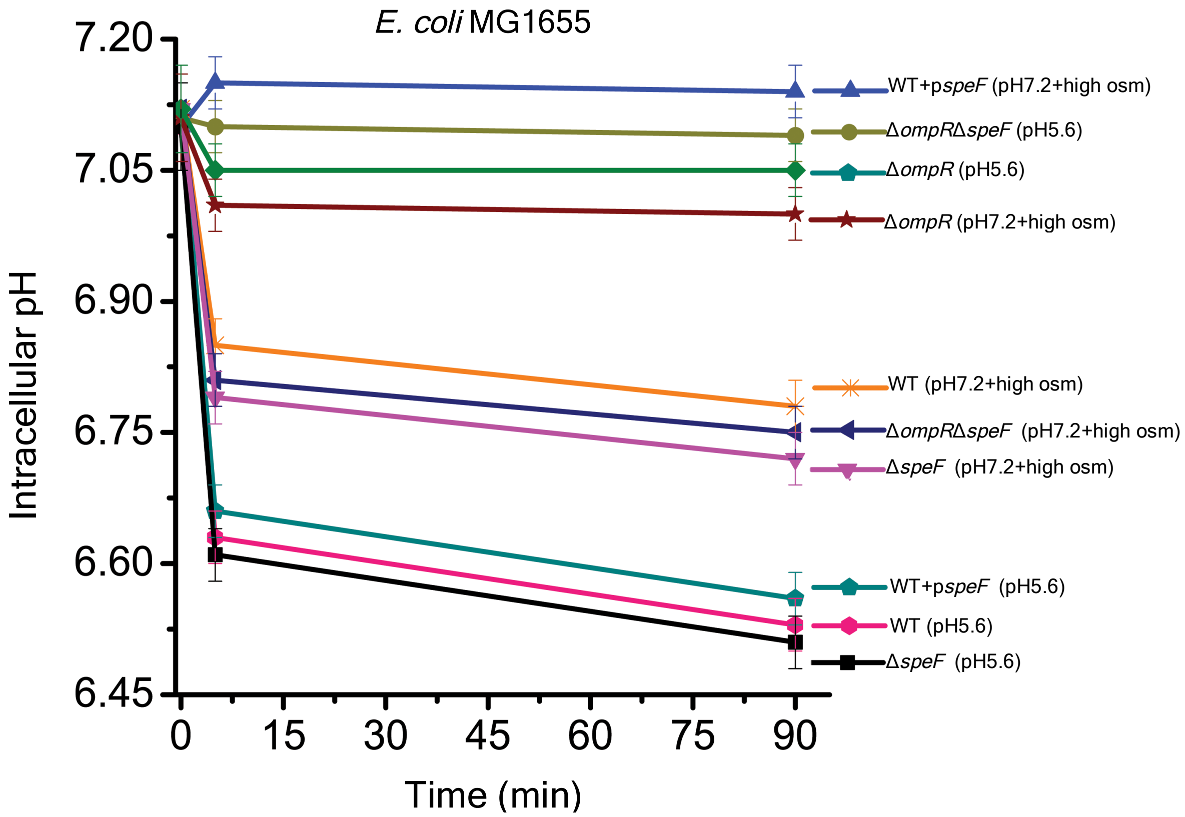

Supplementary Figure 6. At high osmolality, OmpR represses *speF* to acidify the *E. coli* cytoplasm. Plot of intracellular pH measurements of fifty cells of wild-type, an *ompR* null mutant, an *speF* null strain, an *ompR*/*speF* null strain, and an *speF* over-expressed strain of *E. coli* grown at pH_e 5.6 or pH_e 7.2 plus 15% sucrose. Error bars represent the mean ± s.e.m (n = 3).

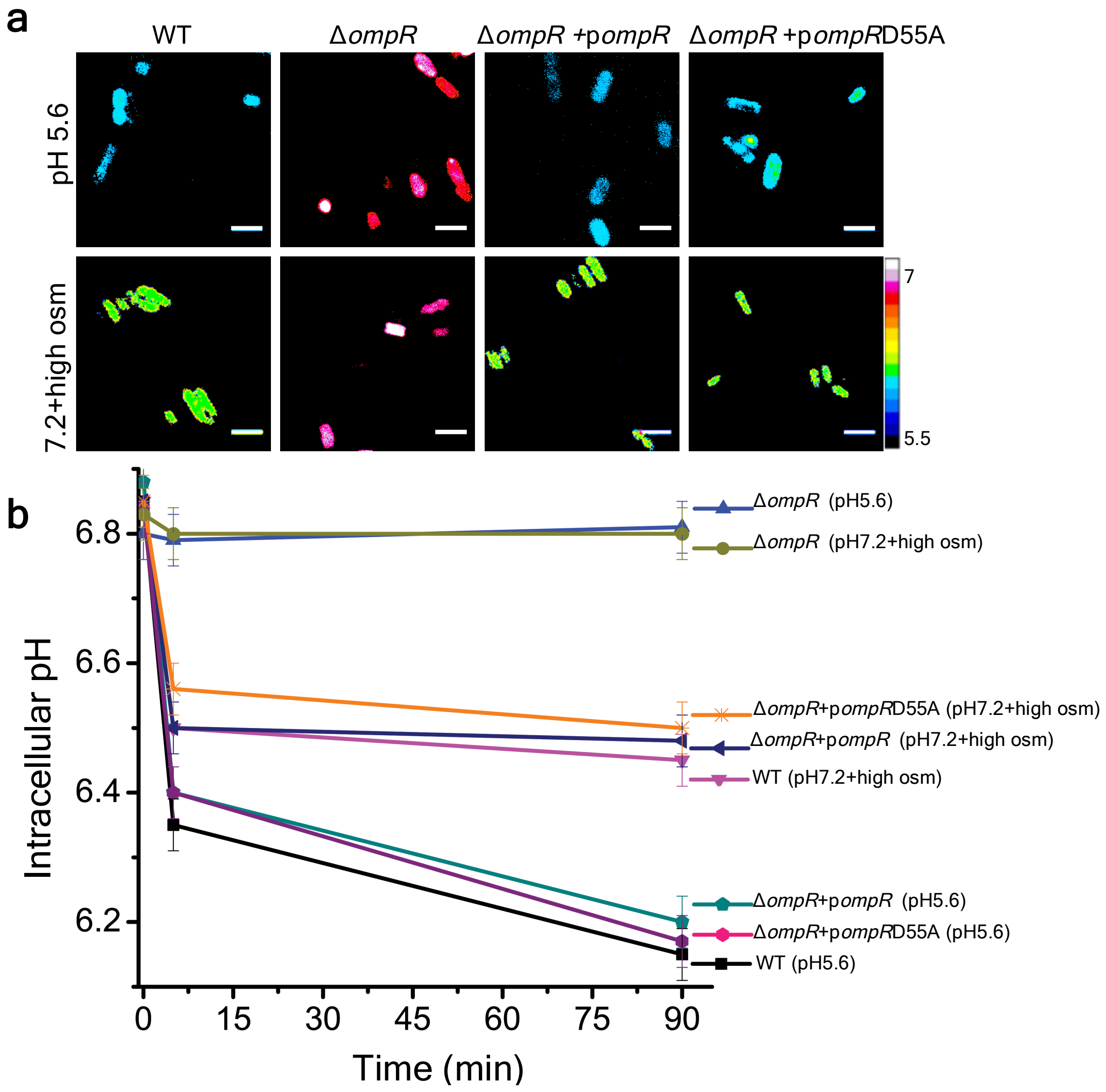
a
WT
ΔompR
ΔompR +pompR
ΔompR +pompRD55A
pH 5.6
7.2+high osm
7
5.5
b
Intracellular pH
6.8
6.6
6.4
6.2
0
15
30
45
60
75
90
Time (min)
ΔompR (pH5.6)
ΔompR (pH7.2+high osm)
ΔompR+pompRD55A (pH7.2+high osm)
ΔompR+pompR (pH7.2+high osm)
WT (pH7.2+high osm)
ΔompR+pompR (pH5.6)
ΔompR+pompRD55A (pH5.6)
WT (pH5.6)

Supplementary Figure 7. OmpR-mediated cytoplasmic acidification does not require phosphorylation. **a,** Representative images of $R_{480/440}$ are shown after 90 min incubation at pH_e 5.6 or pH_e 7.2 plus 15% sucrose of wild-type, an *ompR* null strain and an *ompR* null complemented with either wild-type OmpR or OmpRD55A. Scale bar, 3 μm. **b,** A plot from fifty cells of the $R_{480/440}$ ratios of various mutants of *S***.** Typhimurium at indicated times. Symbols represent the mean ± s.e.m (n= 3).

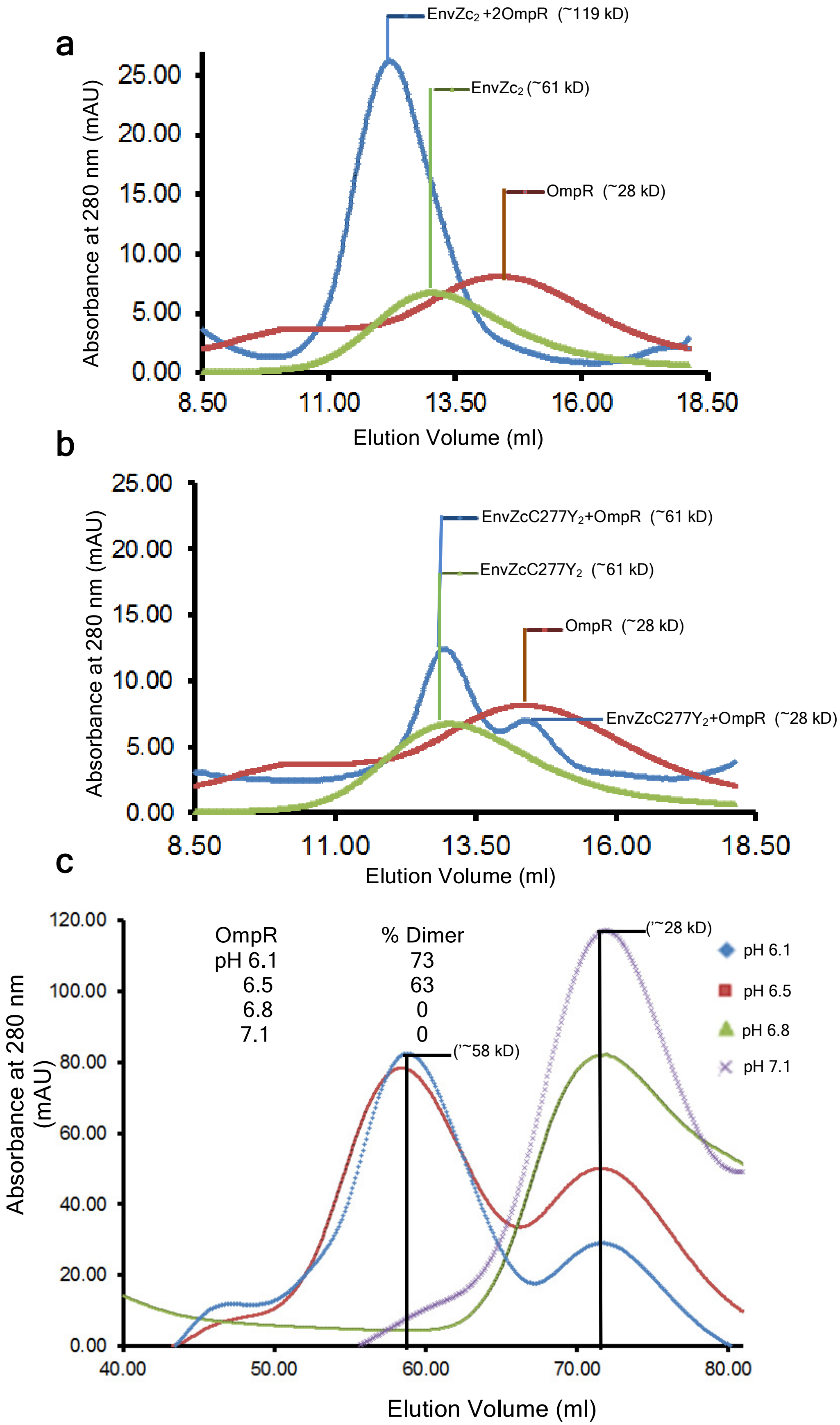
a
EnvZc2 +2OmpR (~119 kD)
EnvZc2 (~61 kD)
OmpR (~28 kD)
Absorbance at 280 nm (mAU)
Elution Volume (ml)
b
EnvZcC277Y2+OmpR (~61 kD)
EnvZcC277Y2 (~61 kD)
OmpR (~28 kD)
EnvZcC277Y2+OmpR (~28 kD)
Absorbance at 280 nm (mAU)
Elution Volume (ml)
c
OmpR % Dimer
pH 6.1 73
6.5 63
6.8 0
7.1 0
(~58 kD)
(~28 kD)
pH 6.1
pH 6.5
pH 6.8
pH 7.1
Absorbance at 280 nm (mAU)
Elution Volume (ml)

Supplementary Figure 8. EnvZc cysteine 277 mutant fails to interact with OmpR. An overlay of the gel filtration profiles of **a,** EnvZc, OmpR and (EnvZc + OmpR) and **b,** (EnvZc C277Y+ OmpR) along with individual EnvZc C277Y and OmpR from a Superose® 12 10/300 (Sigma Aldrich) size exclusion chromatography column buffered at pH 7 (AKTA system, Amersham Biosciences) is shown. EnvZc C277Y doesn't bind to OmpR, as evident from the individual EnvZc C277Y and OmpR peaks, instead of a higher molecular weight shifted complex. **c,** An overlay of the gel filtration profiles of OmpR alone buffered at pH 6.1, pH 6.5, pH 6.8 and pH 7.1 from a Hiload 16/60 Superdex 75 pg (Amersham Biosciences) size exclusion chromatography column on an AKTA system (Amersham Biosciences) is shown.

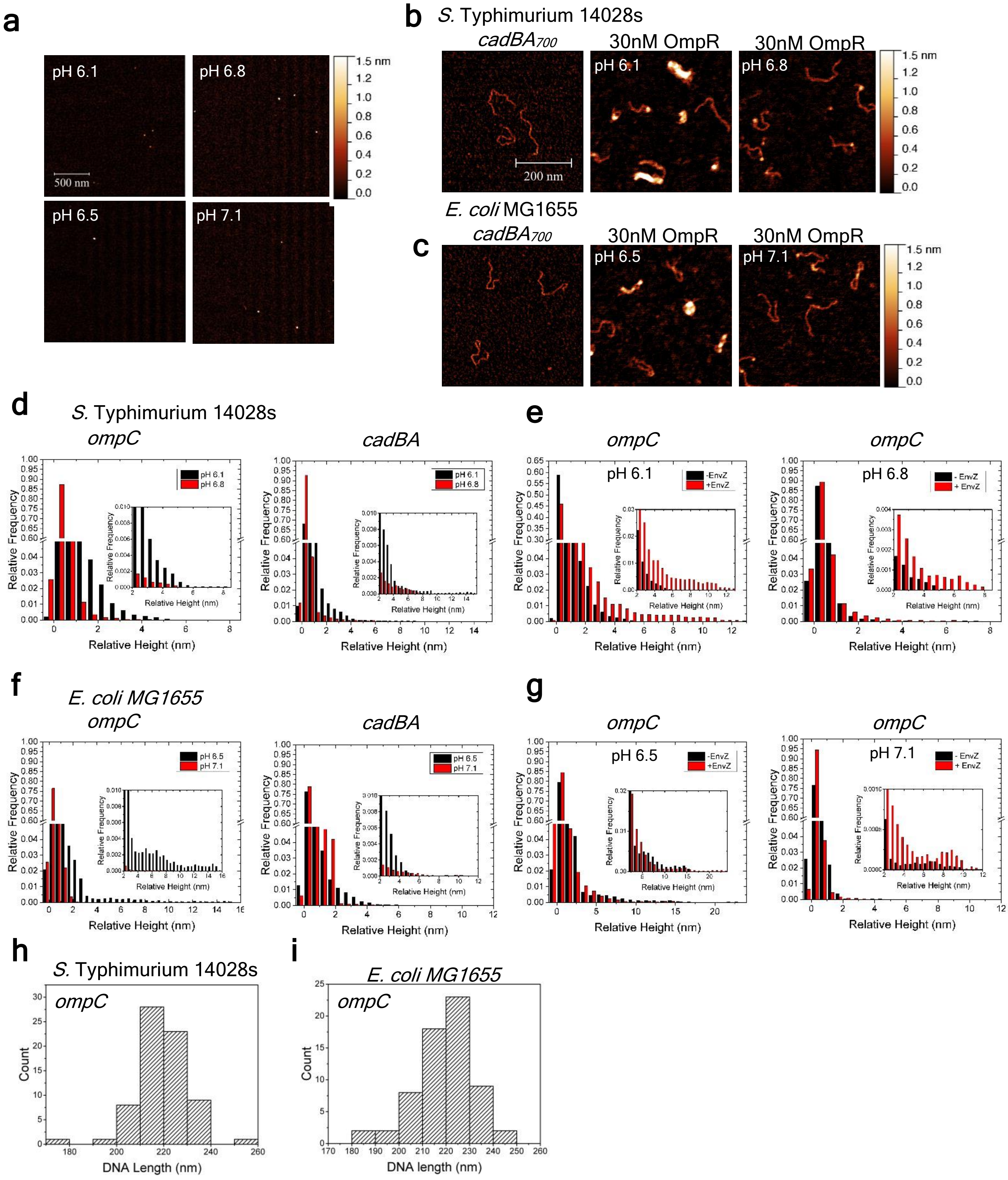

a
pH 6.1
pH 6.8
pH 6.5
pH 7.1
500 nm
1.5 nm
b
S. Typhimurium 14028s
cadBA700
30nM OmpR
200 nm
c
E. coli MG1655
d
ompC
cadBA
pH 6.1
pH 6.8
Relative Frequency
Relative Height (nm)
e
-EnvZ
+EnvZ
f
pH 6.5
pH 7.1
g
h
Count
DNA Length (nm)
i
DNA length (nm)

Supplementary Figure 9. **OmpR shows increased affinity to DNA at acidic pH. a,** AFM images of 30 nM OmpR (no DNA) deposited on mica at various pH values as indicated. AFM images of *cadBA* complexed with 30 nM OmpR at either acidic or neutral pH for **b,** *S.* Typhimurium and **c,** *E. coli*. Relative height distribution histograms of *ompC* and *cadBA* promoters complexed with 30 nM OmpR at either acidic or neutral pH **d,** *S.* Typhimurium and **f,** *E. coli*. Relative height distribution histograms of the *ompC* promoter region with 30 nM OmpR in the presence or absence of 30 nM EnvZ at either acidic or neutral pH for **e,** *S.* Typhimurium and **g,** *E. coli*. The insets (d, e, f, g) show the distribution histograms of relative height > 2 nm, which corresponds to the height of DNA bound with OmpR. For comparison, the relative height of naked DNA is typically 0.5-1 nm in our experimental conditions. The term 'relative height' is used, as the apparent heights measured by AFM do not represent the true height [1]. Acidic pH or addition of EnvZ increases the proportion of the DNA-OmpR protein complex with greater relative height, indicating stronger OmpR binding. Length measurements of the *ompC* promoter from **h,** *S.* Typhimurium and **i,** *E. coli* from images acquired by AFM. The DNA contours were obtained semi-automatically and the contour lengths were obtained with corner count estimator [2]. The measured DNA length was 219 ± 11 nm for *S.* Typhimurium *ompC*, corresponding to 646 ± 31 bp, and 220 ± 13 nm for *E. coli ompC*, corresponding to 646 ± 37 bp.

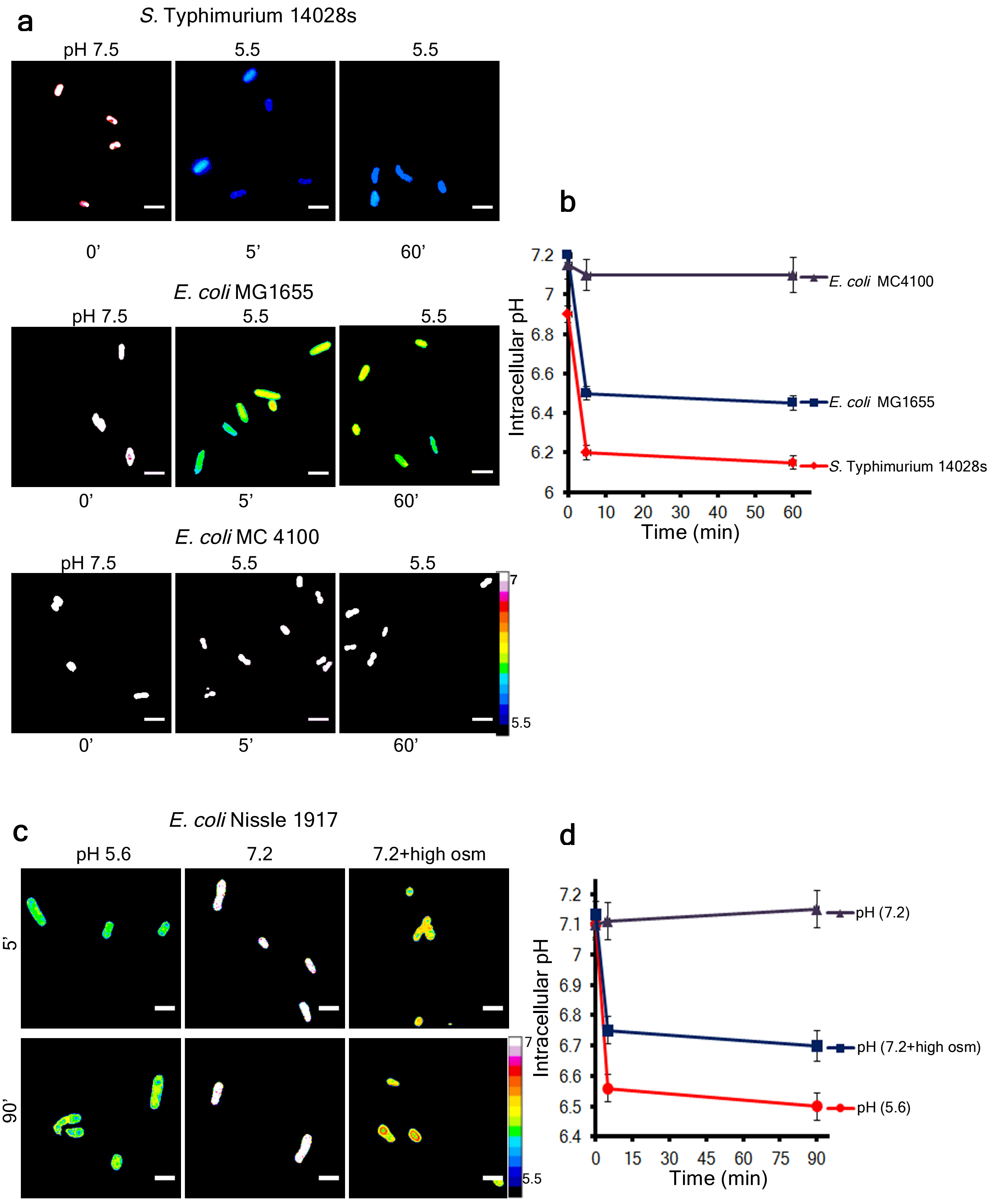
a
S. Typhimurium 14028s
pH 7.5
5.5
5.5
0'
5'
60'
E. coli MG1655
pH 7.5
5.5
5.5
0'
5'
60'
E. coli MC 4100
pH 7.5
5.5
5.5
7
5.5
0'
5'
60'
b
Intracellular pH
7.2
7
6.8
6.6
6.4
6.2
6
0
10
20
30
40
50
60
Time (min)
E. coli MC4100
E. coli MG1655
S. Typhimurium 14028s
c
E. coli Nissle 1917
pH 5.6
7.2
7.2+high osm
5'
90'
7
5.5
d
Intracellular pH
7.2
7.1
7
6.9
6.8
6.7
6.6
6.5
6.4
0
15
30
45
60
75
90
Time (min)
pH (7.2)
pH (7.2+high osm)
pH (5.6)

Supplementary Figure 10. *E. coli* strain Nissle 1917 acidifies its cytoplasm comparable to *E. coli* MG1655 and *S*. Typhimurium. **a,** Representative $R_{480/440}$ images of wild-type strains of *S*. Typhimurium, *E. coli* MG1655 and *E. coli* MC4100 cultured in M63 media supplemented with casein hydrolysate buffered at pH 7.5 are shown after 5 and 60 min post-shift. Scale bar, 3 µm. **b,** Plot of the $R_{480/440}$ ratios of *S*. Typhimurium and *E. coli* strains were determined at each indicated time points. Error bars represent mean ± s.e.m (n = 3). **c,** Representative epifluorescence ratio images ($R_{480/440}$) were obtained for wild-type *E. coli* Nissle1917 strain incubated at pH_e 5.6, pH_e 7.2 and pH_e 7.2 with 15% sucrose at the indicated time points. Scale bar, 3 µm. **d,** A plot of the intracellular pH of fifty cells incubated under acid and osmotic stress conditions at each indicated time points. Error bars represent mean ± s.e.m (n = 3).

Supplementary Table 1. Primers used in this study

**Name**	**Sequences (5' to 3')**
speF::*tetRA* #1F	AATTGAGGACCTGCTATTACCTAAAATAAAGAGATGAAAATTAAGACCC ACTTTCACATT
speF::*tetRA* #1R	GTTAACTGAACGACGCCCATTTTGTTCGATTTAGCCTGACCTAAGCACTT GTCTCCTG
cadBA1655::*tetRA* #1F	ACCCGGACTCCAAATTCAAAAATGAAATTAGGAGAAGAGCTTAAGACC CACTTTCACATT
cadBA1655::*tetRA* #1R	AAAAAGGGAAGTGGCAAGCCACTTCCCTTGTACGAGCTAACTAAGCACT TGTCTCCTG
rpoS::*tetRA* #1F	TTGCTAGTTCCGTCAAGGGATCACGGGTAGGAGCCACCTTTTAAGACCC ACTTTCACATT
rpoS::*tetRA* #1R	AAGGCCAGTCGACAGACTGGCCTTTTTTTGACAAGGGTACCTAAGCACT TGTCTCCTG
yhgA::*tetRA* #1F	TCTACGCTTAAAGGGTGTTCAACCTGAAGGGAGAAGCTCATTAAGACCC ACTTTCACATT
yhgA::*tetRA* #1R	TTGCTTAACAGAATTGCGCCACCTTAATCGACTTCATGCGCTAAGCACTT GTCTCCTG
yhgA::*Cm* #1F	TCTACGCTTAAAGGGTGTTCAACCTGAAGGGAGAAGCTCACATATGAAT ATCCTCCTTA
yhgA::*Cm* #1R	TTGCTTAACAGAATTGCGCCACCTTAATCGACTTCATGCGTGTGTAGGCT GGAGCTGCTTC
D55AF	CATCTCATGGTACTGGCTTTAATGCTGCCAGGT
D55AR	ACCTGGCAGCATTAAAGCCAGTACCATGAGATG
C277YF	TAAAGACATCGAAGAGTATAACGCCATTATCGAAC
C277YF	GTTCGATAATGGCGTTATACTCTTCGATGTCTTTA
speF # F	Bio/CACATCAGGAAAGGTAAATTA
speF # R	Bio/TTTCATCTCTTTATTTTAGGT
rpoS # F	Bio/CTGGCCGACGGATGGCAAAGTG
rpoS # R	Bio/AAGGTGGCTCCTACCCGTGA
cadA # F	Bio/ TCGGTGAACTGACCGGTATCG
cadA # R	Bio/ CAATAACGTTCATAGTCATA
cadB # F	Bio/CATCAGGAGCGTAACGCATCC
cadB # R	Bio/GCTCTTCTCCTAATTTCATTT
yghA # F	Bio/ TATGCGTTACAGGTCAAAGAG
yghA# R	Bio/ AGACATTGAGCTTCTCCCTTC
NcoI-*cadBA* #1F	CATGCCATGGGAA GTTCTGCCAAGAAGATCGG
cadBA-XbaI #1R	GCTCTAGATTATTTTTTGCTTTCTTCTTTCA
NcoI-*gadBA* #1F	CATGCCATGGATGGATAAGAAGCAAGTAACGG
gadBA-XbaI #1R	GCTCTAGATCAGGTGTGTTTAAAGCTGTTC
NcoI-*speF* #1F	CATGCCATGG CATGTCAAAATTAAAAATTGCGGTT
speF-XbaI #1R	CTAGTCTAGACTAG TCATAATTTTTCCCCTTTCA

NcoI-*speD* #1F	CATGCCATGGCATG ATGAAAAAACTGAAACTGCATG
speD-XbaI #1R	CTAGTCTAGACTAGTTAAACAGCTGGCATATTGCGC
NcoI-*speA* #1F	CATGCCATGGATGTCTGACGACATGTCTATG
speA-XbaI #1R	TACTTATCTTGAAGATGAGTAA
NcoI-*rpoS* #1F	CATGCCATGG ATGAGTCAGAATACGCTGAAA
rpoS-XbaI #1R	CATGCCATGG CATG TCTCATCTATACGACCCTAC
NcoI-*yghA* #1F	GCTCTAGATTAGCCTAAATGCTCCCCGCC
yhgA-XbaI #1R	CCCAAGCTTCATTCCCCTCCATATACACG-
EcoR1-*phluorin* #1F	GGCCGAATTCACCATGAGTAAAGGAGAAGAACT
phluorin-HindIII #1R	GGCCAAGCTT TTATTTGTATAGTTCATCCA
ompC 1655 F	AATAGATGTACAAGCGCCAT
ompC 1655 R	TTTGTTGCCGTCTTTGTTGTAAAC
ompC 14028s F	GAAAAGCGTACAACCTTCGCA
ompC 14028s R	TTTGTTGCCGTCTTTATTATA

Supplementary References

1. Lai, C.-Y., Santos, S. & Chiesa, M. General interpretation and theory of apparent height in dynamic atomic force microscopy. *RSC Adv.* **5,** 80069–80075 (2015).
2. Rivetti, C. & Codeluppi, S. Accurate length determination of DNA molecules visualized by atomic force microscopy: Evidence for a partial B-to A-form transition on mica. *Ultramicroscopy* **87,** 55–66 (2001).
